# P2X7 purinergic receptor plays a critical role in maintaining T-cell homeostasis and preventing lupus pathogenesis

**DOI:** 10.3389/fimmu.2022.957008

**Published:** 2022-09-29

**Authors:** Amine Mellouk, Tom Hutteau-Hamel, Julie Legrand, Hanaa Safya, Mohcine Benbijja, Françoise Mercier-Nomé, Karim Benihoud, Jean M. Kanellopoulos, Pierre Bobé

**Affiliations:** ^1^ UMR 996, INSERM, Université Paris-Saclay, Clamart, France; ^2^ Institut André Lwoff, CNRS, Université Paris-Sud, Villejuif, France; ^3^ Plateforme d’Histologie Immunopathologie de Clamart, IPSIT, INSERM, CNRS, Université Paris-Saclay, Châtenay-Malabry, France; ^4^ UMR 9018, Institut Gustave Roussy, CNRS, Université Paris-Saclay, Villejuif, France; ^5^ Institute for Integrative Biology of the Cell (I2BC), CEA, CNRS, Université Paris-Saclay, Gif-sur-Yvette, France

**Keywords:** SLE (systemic lupus erythematosus), ALPS (autoimmune lymphoproliferative syndrome), p2x7 (purino) receptor, fas (APO-1/CD95), *lpr* mice, T cell, cell death

## Abstract

The severe lymphoproliferative and lupus diseases developed by MRL/*lpr* mice depend on interactions between the Fas^*lpr*^ mutation and MRL genetic background. Thus, the Fas^*lpr*^ mutation causes limited disease in C57BL/6 mice. We previously found that accumulating B220^+^ CD4^–^CD8^–^ double negative (DN) T cells in MRL/*lpr* mice show defective P2X7 receptor ( P2X7)-induced cellular functions, suggesting that P2X7 contributes to T-cell homeostasis, along with Fas. Therefore, we generated a B6/*lpr* mouse strain (called B6/*lpr*-*p2x7*KO) carrying homozygous P2X7 knockout alleles. B6/*lpr*-*p2x7*KO mice accumulated high numbers of FasL-expressing B220^+^ DN T cells of CD45RB^high^CD44^high^ effector/memory CD8^+^ T-cell origin and developed severe lupus, characterized by leukocyte infiltration into the tissues, high levels of IgG anti-dsDNA and rheumatoid factor autoantibodies, and marked cytokine network dysregulation. B6/*lpr*-*p2x7*KO mice also exhibited a considerably reduced lifespan. P2X7 is therefore a novel regulator of T-cell homeostasis, of which cooperation with Fas is critical to prevent lymphoaccumulation and autoimmunity.

## Introduction

Mutations of the Fas death receptor gene cause autoimmune lymphoproliferative syndrome (ALPS) (1). Fas-deficient MRL/*lpr* mice spontaneously develop ALPS and a lupus-like disease, characterized by elevated serum levels of autoantibodies and cytokines, skin rash, arthritis, vasculitis, and nephritis (2). Impaired Fas function in effector CD4 or CD8 single-positive (SP) T cells causes the accumulation of B220^+^ CD4^–^CD8^–^ double-negative (DN) TCRαβ^+^ T cells and, ultimately, splenomegaly and lymphadenopathy (1). A DN T-cell subset that is mainly derived from CD8^+^ precursors also expands in the peripheral blood and disease-affected kidneys of systemic lupus erythematosus (SLE) patients (3, 4). B cells are also critical for full-blown ALPS. Fas deficiency in germinal-center B cells and the resulting memory B cells leads to the accumulation of autoreactive B cells, whereas Fas restoration on *lpr* B cells significantly reduces disease severity (5). Furthermore, T and B cells from MRL/*lpr* mice markedly overexpress Fas ligand (FasL), which renders them cytotoxic in vitro (6, 7) and in vivo (8, 9). As the *lpr* mutation is leaky, FasL overexpression could cause autoimmune attack on Fas^low^-expressing tissues. Aside from the *lpr* mutation, unidentified genes in the MRL genetic background contribute to disease severity, as Fas-deficient C57BL/6 mice (B6/*lpr*) develop limited disease (10).

Numerous susceptibility loci have been discovered in SLE patients and mouse models (2, 11), including the purinergic receptor P2X7 (12, 13). The adenosine triphosphate (ATP)-gated cation channel P2X7 plays a major role in various immune processes, such as the maturation of proinflammatory cytokines (14), the proteolytic cleavage of ectodomains (15, 16), and the activation of T cells (16). Moreover, P2X7 activation can induce the differentiation of conventional and regulatory CD4+ T cells into proinflammatory Th17 cells during chronic inflammation (17–20). However, both detrimental and protective effects of P2X7 on autoimmune diseases have been reported (21–25). We previously showed that the regulation of ATP sensitivity in normal CD4 and CD8 SP T cells depends on their stage of activation and differentiation (26, 27). Moreover, we found that B220^+^ DN T cells that accumulate in MRL/*lpr* and B6/*lpr* mice exhibit a large reduction in P2X7 membrane expression and sensitivity to ATP (28), suggesting that the ATP/ P2X7 pathway may contribute to T-cell homeostasis, together with the Fas apoptotic pathway. Here, we therefore generated a B6/*lpr* mouse strain (called B6/*lpr*-*p2x7*KO) carrying homozygous P2X7 knockout alleles. During ageing, B6/*lpr*-*p2x7*KO mice showed massive accumulation of FasL-expressing B220^+^ DN T cells in the periphery, leading to marked splenomegaly, lymphadenopathy, and hepatomegaly. B6/*lpr*-*p2x7*KO sera contained high levels of IgG anti-double-stranded (ds)DNA antibodies and rheumatoid factor (RF), the proinflammatory cytokines IFN-λ, IL-1β, IL-6, TNF-α, IL-15/IL-15Rα complex, IL-17A, and IL-23, and the B-cell survival factors IL-10 and BAFF, but only low levels of the anti-inflammatory cytokine TGF-β1. Overall, our data strongly suggest that the P2X7 deficiency strongly amplified the mild phenotype of B6/*lpr* mice, as lymphoaccumulation, autoantibody production, and dysregulated cytokine/chemokine expression were not found in P2X7-deficient B6 mice. Finally, B6/*lpr*-*p2x7*KO mice showed a remarkably shortened lifespan, with 50% cumulative mortality at 20 weeks of age for female and male mice. This study reveals an unanticipated protective role for P2X7 in T-cell homeostasis and the development of ALPS and SLE.

## Materials and methods

### Mice

Fas deficient MRL/MpJ-Fas^*lpr*^/J (MRL/*lpr*, stock No. 000485), wildtype C57BL/6J (B6), P2X7-deficient B6 (B6-*p2x7*KO, stock No. 005576), and Fas-deficient B6 (B6/*lpr*, stock No. 000482) mouse strains were obtained from The Jackson Laboratory (Bar Harbor, ME). The B6/*lpr*-*p2x7*KO mouse strain homozygous for both the fas^lpr^ (lpr) mutation and P2X7 knockout allele was generated as follows: B6/*lpr* and B6-*p2x7*KO mice were mated to produce F1 hybrid mice uniformly heterozygous for the *lpr* mutation and P2X7 knockout allele. Interbreeding of these F1 mice allowed the production of F2 mice (called B6/*lpr*-*p2x7*KO) homozygous for the *lpr* mutation and P2X7 knockout allele. All mouse strains were maintained in our animal facilities (CNRS TAAM UPS44, Orléans, France, and Plateforme Hébergement et Expérimentation Animale BUFFON, Paris, France). Although male and female B6/*lpr*-*p2x7*KO mice develop ALPS and lupus during ageing, the analysis of female mice were favoured in each experimental group unless indicated otherwise because of the significant female sex bias in lupus patients and lupus-prone MRL/*lpr* mice. Animal protocols were approved by local (CEEA03 and CEEA40) and national (MESRI) ethics committees for research (agreement number: D452346).

### PCR-based genotyping analysis of B6/*lpr*-*p2x7*KO double-mutant mice

F1 and F2 generations of B6/*lpr* and B6-*p2x7*KO intercrosses were screened for the presence of wildtype or mutant alleles of P2X7 and *fas* genes by PCR using DNA extracted from the tails and forward 5’-TCACCACCTCCAAGCTCTTC-3’ and reverse 5’-TATACTGCCCCTCGGTCTTG-3’ primers for the P2X7 gene, forward 5’-GTAAATAATTGTGCTTCGTCAG-3’ and reverse 5’-CAAATCTAGGCATTAACAGTG-3’ primers for the *fas* gene, and forward 5’-GTAAATAATTGTGCTTCGTCAG-3’ and reverse 5’-TAGAAAGGTGCACGGGTGTG-3’ primers for insertion of the early transposon (ETn) into the fas gene (*lpr* mutation). Cycling conditions were as follows: 1 cycle at 95°C for 5 min; 30 cycles at 95°C for 1 min, 60°C for 30 s, and 72°C for 45 s; and 1 cycle at 72°C for 5 min.

### Single-cell suspension preparations

The single-cell suspensions were prepared from whole organs. Spleens, lymph nodes (inguinal and cervical) and thymus were mechanically dissociated in 5% fetal calf serum (FCS)-RPMI 1640 medium (Gibco, ThermoFisher Scientific) and passed through a 70 µm nylon cell strainer to obtain uniform single-cell suspensions. Bone marrow cells were flushed from the femur and tibia of one hind leg with 5% (FCS)-RPMI 1640 medium, passed through a nylon cell strainer and washed. Intrahepatic lymphocytes (IHL) were isolated as previously described (8). Briefly, whole livers were perfused to eliminate blood, cut into small pieces in 30% FCS-RPMI 1640 medium (Gibco, ThermoFisher Scientific), passed through a mesh, and washed. The cell pellet was resuspended in 20% FCS-RPMI 1640 medium and mononuclear cells were separated from hepatocytes by Ficoll density (1,090 g/ml) gradient centrifugation.

### ATP-mediated cellular function assays

Assays for P2X7-mediated CD62L shedding, phosphatidylserine exposure, and pore formation were performed as previously described (26, 27). Briefly, spleen cells (10^6^ cells/ml) suspended in RPMI 1640 were treated with 500 µM ATP in a humidified 5% CO2 atmosphere at 37°C for 30 min. Then, cell suspensions were washed and stained for 30 min on ice with phenotype-specific fluorescent mAbs and fluorescent-conjugated anti-CD62L mAb and Annexin V fluorescent conjugates to assess CD62L shedding and cell-surface exposure of phosphatidylserine (PS). To quantify P2X7-mediated pore formation, ATP treatment was performed in the presence of YO-PRO-1 or -3 fluorescent dyes (Thermo Fisher Scientific).

### Lymphocyte immunophenotyping

Single-cell suspensions prepared from whole organs were phenotyped by flow cytometry using fluorescent-conjugated monoclonal antibodies (mAb) directed against the cell surface markers CD90.2/Thy1.2 (clone 30-H12), B220 (clone RA3-6B2), CD19 (clone 1D3), IgM (clone II/41), IgD (clone 11-26), CD45RB (clone C363.16A), CD4 (clone GK1.5), CD8α (clone 53-6.7), CD69 (clone H1.2F3), CD44 (clone IM7), CD62L (clone MEL-14), CD25 (PC61.5), CTLA-4 (clone UC10-4B9), CD197/CCR7 (clone 4B12), KLRG1 (clone 2F1), CD127/IL-7 receptor-α (clone A7R34), PD-1 (clone J43), and Tim-3 (clone 2B.2C12), the death-inducing molecules FasL (clone MFL3), Fas (clone Jo2), and Granzyme B (clone NGZB), and the transcription factors Foxp3 (clone FJK-16s) and Eomesodermin (clone Dan11mag) (eBioscience, ThermoFisher Scientific or BD Biosciences). P2X7 was detected using a rabbit polyclonal anti- P2X7 serum and fluorescent-conjugated goat anti-rabbit IgG F(ab)’2 secondary antibodies (eBioscience), as described previously (26–28). Fluorescent-conjugated rat or mouse IgG1, IgG2a, or IgG2b, Armenian hamster IgG mAbs, or rabbit IgG polyclonal serum were used as isotype controls (eBioscience). The use of a mAb to the mouse Fcγ receptor (clone 93, eBioscience) avoided non-specific antibody binding. At least 20,000 events were analyzed for each sample. Cell debris, dead cells, and doublets were gated out using the FSC and SSC parameters. Data acquisition was performed at the Flow Cytometry Core Facility at I2BC (Gif-sur-Yvette, France) and IPSIT (Clamart, France). Flow cytometry data were analyzed using Summit (Beckman Coulter) and FlowJo (Treestar) software. The immunophenotyping of the different CD4 or CD8 single-positive (SP) and B220^+^ CD4^–^CD8^–^ double-negative (DN) CD90^+^ T-cell subsets have been identified and gated using a sequential gating strategy (**Figure S1**).

### Quantification of anti-dsDNA antibody, RF, cytokine, and soluble FasL levels

Serum levels of IgG anti-dsDNA autoantibodies were quantified using an anti-dsDNA ELISA kit (Signosis), according to the manufacturer’s instructions. Serum levels of IgG2a anti-dsDNA and IgG-RF autoantibodies were quantified by ELISA as previously described (29). Briefly, 96-well plates were coated either with calf-thymus dsDNA or rabbit IgG (Sigma-Aldrich) and blocked with 1% bovine serum albumin solution. Serial serum dilutions were added in triplicate and the plates incubated at room temperature for 2 h and then washed. Bound IgG2a anti-dsDNA and IgG RF were detected using alkaline phosphatase-labelled goat anti-mouse IgG2a and IgG, respectively, and para-nitrophenyl phosphate (pNPP, Sigma-Aldrich). The OD was determined at 405 nm. Serum levels of MCP-1/CCL2, MIP-1α/CCL3, MIP-1β/CCL4, RANTES/CCL5, MCP-3/CCL7, Eotaxin/CCL11, GROα/CXCL1, MIP-2/CXCL2, ENA-78/CXCL5, IP-10/CXCL10, BAFF, IL-1α, IL-1β, IL-2, IL-3, IL-4, IL-5, IL-6, IL-9, IL-10, IL-12 p70, IL-13, IL-15/IL-15R complex, IL-17A, IL-18, IL-22, IL-23, IL-27, IL-28/IFN-λ, IL-31, LIF, M-CSF, G-CSF/CSF-3, GM-CSF, IFN-α, IFN-γ, TGF-β1, TNF-α, and soluble FasL were assayed using the cytokine & chemokine 36-Plex Mouse ProcartaPlex Kit, TGF beta 1 Mouse Simplex ProcartaPlex Kit (eBioscience, Thermo Fisher Scientific), Mouse BAFF/BLyS/TNFSF13B, Mouse/Rat IL-33 and the Mouse Fas Ligand/TNFSF6 Quantikine ELISA Kits (R&D systems, Minneapolis, MN), following the manufacturers’ instructions.

### Determination of biochemical parameters

Serum urea, aspartate aminotransferase (ASAT), and lactate dehydrogenase (LDH) levels were quantified using an automated analyser (Olympus AU400 Clinical Chemistry Analyzer, Biochemistry laboratory facility, UMR1149 INSERM-Université Paris Diderot, France).

### Histopathological and immunohistofluorescence analyses

Kidney, liver, and lung samples were fixed overnight with zinc formalin fixative (Sigma-Aldrich) and then embedded in paraffin. Tissue sections (5 µm) were stained with hematoxylin and eosin, Picro-Sirius red, or Masson’s trichrome (Microm-Microtech; France). Slides were scanned using a NanoZoomer 2.0-RS digital slide scanner and 2×, 10×, or 30× objective lenses with a numerical aperture of 0.75 (Hamamatsu Photonics), and images were analyzed using NDP View2 software (Hamamatsu Photonics). Immunohistofluorescence staining was performed on paraffin sections using primary antibodies against the pan-T cell marker CD3 (clone CD3-12, Abcam; 1/250) and the macrophage marker CD68 (PA5-89134, Thermo Fischer Scientific; 1/200), followed by staining with appropriate secondary antibodies Alexa Fluor™ 488 and Alexa Fluor™ 594 (Invitrogen, Thermo Fisher Scientific, 1/100). Nuclei were stained with Hoechst 33342 (Invitrogen; Molecular Probes; 1/300).

### Statistical analysis

Data were expressed as means with standard deviations (SD). A value of more than three SDs from the mean served as criterion to exclude outliers. Data were analyzed using GraphPad Prism 9.4.0 software (La Jolla, CA). The Shapiro-Wilk test was used to assess the normality of data distribution. The two-tailed Student’s t-test for unpaired data was used to compare the mean of two independent groups. Where two independent variables were considered, two-way analysis of variance (ANOVA) with a Tukey post hoc test was used to compare the means of more than two groups. Statistical significance is indicated as follows: ns, not significant; * p ≤ 0.05; ** p ≤ 0.01; *** p ≤ 0.001, **** p ≤ 0.0001.

## Results

### B6/*lpr*-*p2x7*KO mice develop massive lymphadenopathy and splenomegaly

**Figure 1:**
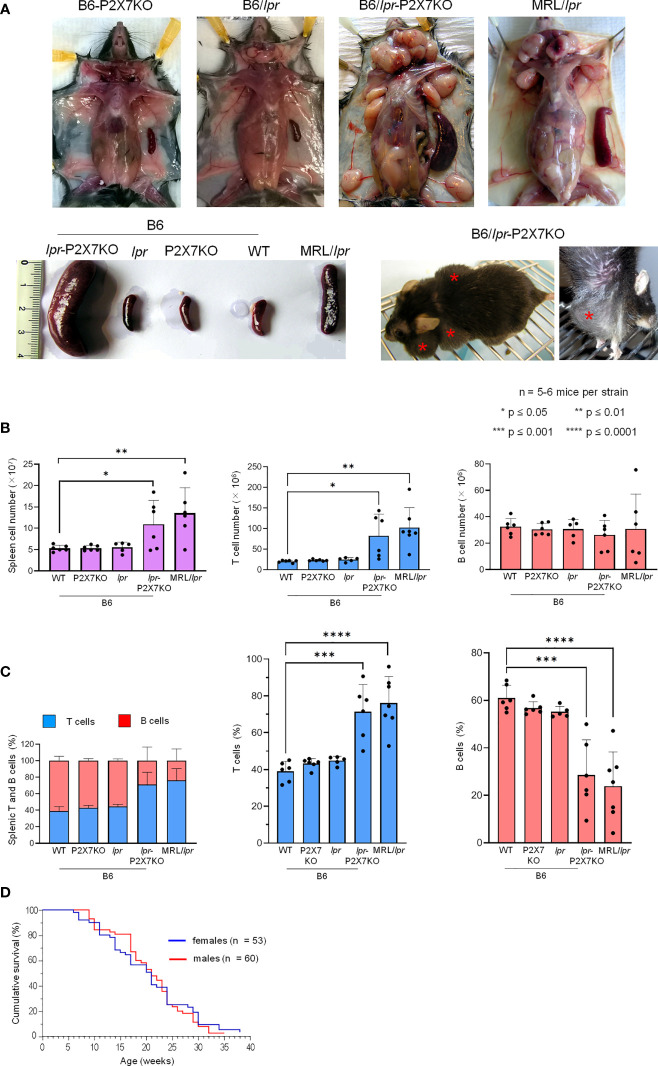
B6/*lpr*-*p2x7*KO mice show a shortened lifespan and develop massive hyperplasia of the secondary lymphoid organs with aging due to the accumulation of T lymphocytes.Representative images of cervical, axillary, and inguinal lymph nodes and spleens **(A)** of 7- to 8-month-old female B6 (WT), B6-*p2x7*KO, B6/ *lpr* and B6/ *lpr* -*p2x7*KO mice and 4-month-old female MRL/ *lpr* mice. The massive hyperplasia of lymph nodes is noticeable in a whole body image of living B6/ *lpr* -*p2x7*KO mice (A, bottom panel, red asterisks). **(B, C)** Spleen cells of 7- to 8-month-old female WT, B6-*p2x7*KO, B6/ *lpr* and B6/ *lpr* -*p2x7*KO mice and 4-month-old female MRL/ *lpr* mice were stained with either fluorescent mAbs against phenotypic markers CD90 and B220 or isotype controls and analyzed by flow cytometry. Bar graphs show the absolute number **(B)** and percentage **(C)** of splenic leukocytes (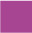), CD90^+^ T cells (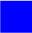) and CD90^−^B220^+^ B cells (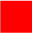) from each mouse strain. Stacked bar graphs show the respective proportions of the T and B cells in each mouse strain. Data are expressed as mean (SD) of 5-6 mice per strain, and each dot in the bar graphs represents an individual mouse. Error bars in graphs show SD. **(D)** Kaplan-Meier graph showing the cumulative survival rate by age for female (n = 53) and male (n = 60) B6*/ *lpr**-*p2x7*KO mice. Asterisks above horizontal lines indicate statistically significant differences assessed with unpaired two-tailed *t*-test. *p ≤ 0.05, **p ≤ 0.01, ***p ≤ 0.001 ****p ≤ 0.0001.

We previously reported that pathogenic B220^+^ DN T cells from MRL/*lpr* or B6/*lpr* mice show a strong reduction of P2X7 membrane expression and sensitivity to extracellular ATP (28). We generated a B6/*lpr* mouse strain carrying homozygous P2X7 knockout alleles (named B6/*lpr*-*p2x7*KO) to determine whether the downexpression of P2X7 on B220^+^ DN T cells contributes to their accumulation in *lpr* mice. B6/*lpr* mice were chosen because they develop only mild lymphoaccumulation relative to MRL/*lpr* mice. B6/*lpr*-*p2x7*KO mice were produced by crossing B6/*lpr* and B6-*p2x7*KO mice. Double-mutant mice were selected in the F2 generation by PCR-based genotyping. The absence of P2X7 and Fas on splenocytes of F2 mice was confirmed by flow cytometry quantification of ATP-induced cellular functions and P2X7 and Fas membrane expression (**Figures S2A, B**). Unlike the parental strains, B6/*lpr*-*p2x7*KO mice developed unexpected massive hyperplasia of the peripheral lymphoid organs comparable to those of MRL/*lpr* mice (**Figures 1A, B**). The weight of the spleen and LNs in six-month-old B6/*lpr*-*p2x7*KO mice was 4 to 6 times higher than in age-matched B6/*lpr* mice (**Table 1**). The increased size of these lymphoid organs was due to increased cell numbers, mostly T cells. Although the absolute B-cell numbers were unchanged in B6/*lpr*-*p2x7*KO mice, their percentage was markedly reduced due to the massive increase in absolute T-cell numbers (**Figures 1B, C**, **S2C**). Moreover, B6/*lpr*-*p2x7*KO mice showed a remarkably reduced lifespan, with 50% cumulative mortality at 20 weeks of age for both male and female mice (**Figure 1D**; **Table 2**). Therefore, B6/*lpr*-*p2x7*KO mice develop a more rapid and severe form of disease than B6/*lpr* mice.

**Table 1:** Organ weights of B6/*lpr*-*p2x7*KO mice compared to parental strains and MRL/ *lpr* mice.

	B6-*p2x7*KO*** (n=7)*	B6*/ *lpr** (n=7)	B6/ *lpr* -*p2x7*KO (n=8)	MRL*/ *lpr** (n=6)
Axillary LN**	0.007 (0.004)	0.054 (0.072)	0.338 (0.228)	0.257 (0.144)
Mesenteric LN	0.064 (0.021)	0.199 (0.226)	0.782 (0.243)	0.902 (0.368)
Spleen	0.090 (0.031)	0.200 (0.085)	0.787 (0.818)	0.880 (0.474)
Liver	1.800 (0.178)	1.790 (0.206)	2.276 (0.587)	2.637 (0.483)

*n corresponds to the number of mice per group; **Organ weight in g. Results are expressed as mean (SD) from 6-8 mice per strain. LN= lymph node; ***7- to 8-month-old B6-*p2x7*KO, B6/ *lpr* and B6/ *lpr* -*p2x7*KO and 4-month-old MRL/ *lpr* mice.

**Table 2:** Summary of the major phenotypic characteristics of B6/ *lpr* -*p2x7*KO mice compared to parental strains and the MRL/ *lpr* mouse model of ALPS and SLE diseases.

Mouse strain		B6	B6-*p2x7*KO	B6/ *lpr*	B6/ *lpr* -*p2x7*KO	MRL/ *lpr*
**50% mortality (months)**		> 24	> 24	14-17	5	4-6
**B220^+^ DN T cell numbers**	**Spleen**	−	−	+	++++	++++
	**Liver**	−	−	−	++++	−
Anti-dsDNA Ab*		4 (2.2)	2 (1.3)	33 (19.4)	168 (52)	151 (78)
**Lymphoid infiltrates**	**Lung**	−	−	−	++++	++++
	**Kidney**	−	−	−	++++	++++
	**Liver**	−	−	−	++++	−
	**Skin**	−	−	−	+/−	++++
**Cytokines**	TGF-β*	6 (2)	5.7 (2.8)	1.6 (1.3)	1.12 (0.6)	1.98 (0.9)
	IL-10**	23 (22.4)	19 (10)	92 (105)	126 (80.8)	93 (68.5)
	IFN-λ**	70 (14.3)	69 (14)	198 (92)	369 (325)	150 (118)
	IL-15/IL-15R**	0.16 (0.5)	0	7.6 (9.5)	33 (42.5)	0.90 (2.8)
	BAFF*	13 (0.5)	13.4 (5.3)	9.5 (0.6)	18 (3.7)	21.8 (1.8)
	IL-6**	33 (6.6)	23 (18)	173 (112)	277 (153)	277 (223)
	IL-1β**	3 (2.2)	5 (4)	18 (11.6)	36 (23.5)	11 (11)
	TNF-α**	6 (2)	6.6 (1.6)	10.3 (3.6)	19 (9.2)	83 (83.6)
	IFN-γ**	8 (5.4)	10 (9.6)	84 (68.7)	103 (125)	36 (30.3)
	IL-17A**	0.3 (0.6)	0.9 (1.1)	4.4 (4.9)	6.2 (6.5)	2 (3.9)
**FasL**	**Soluble**	+	−	+++	+++	++++
	**Transmembrane**	+	+	+	++	+++

+/−, very low level; +, low level; ++, moderate level; +++, high level; ++++, very high level; *ng/ml; **pg/ml, Results are expressed as mean (SD), n = 5-11 mice per strain.

#### Massive accumulation of B220^+^ DN T cells in B6/*lpr*-*p2x7*KO spleens and LNs

**Figure 2:**
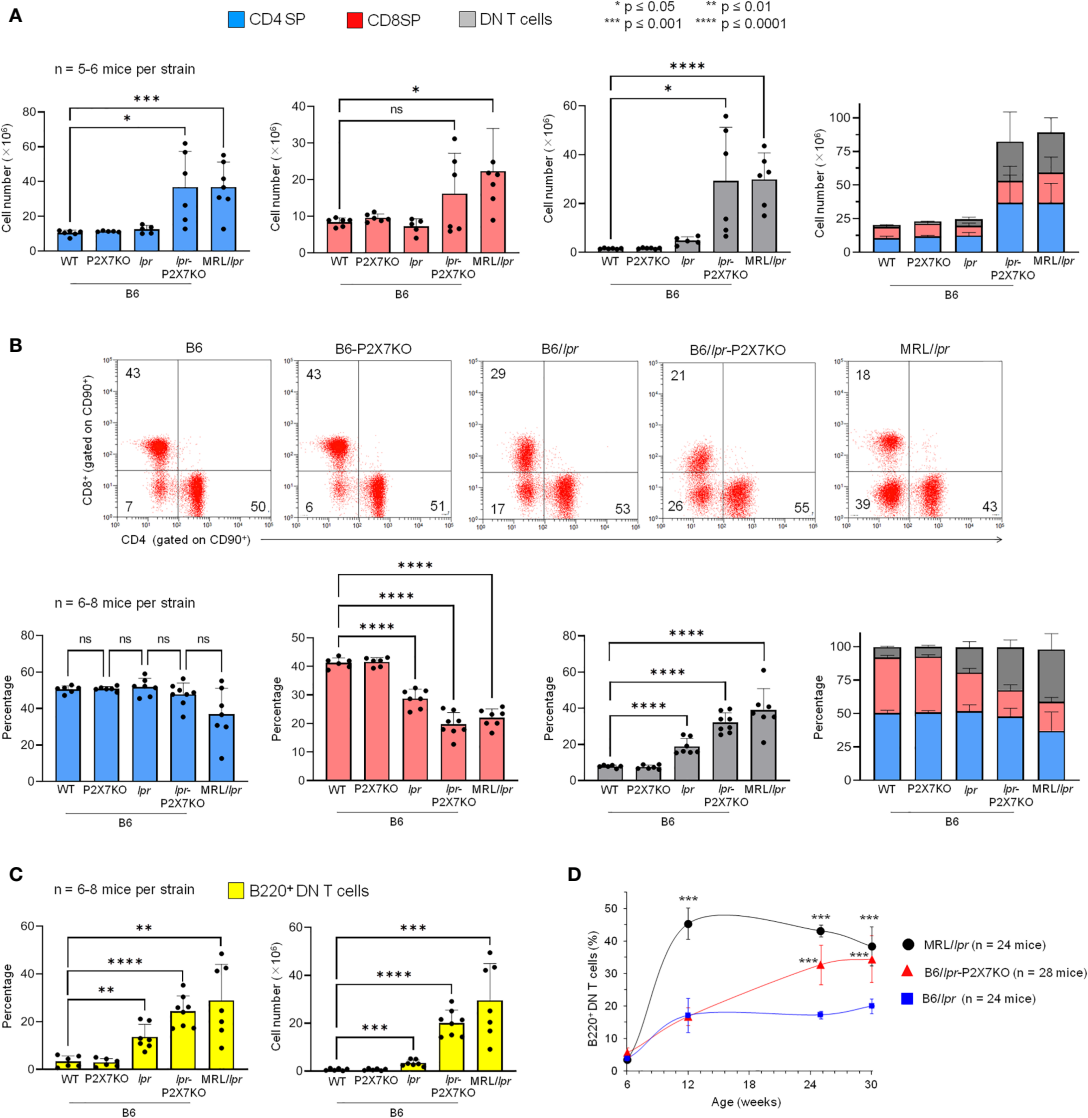
Quantification of the percentage and absolute number of SP, DN, and B220^+^ DN T cells and level of CD4 and CD8 on SP T cells in B6/*lpr* - *p2x7*KO mice and parental strains. Spleen cells from 7- to 8-month-old female B6, B6-*p2x7*KO, B6/ *lpr* and B6/ *lpr* -*p2x7*KO mice and 4-month-old female MRL/ *lpr* mice were stained with either fluorescent mAbs against the phenotypic markers CD90, B220, CD4, and CD8 or isotype controls and analyzed by flow cytometry. Bar graphs show the absolute number **(A)** and percentage **(B)** of CD4 SP (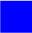), CD8 SP (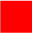) and DN (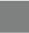) T cells among CD90^+^ T cells from each mouse strain. Data are expressed as mean (SD) of 5-8 mice per strain and each dot in the bar graphs represents an individual mouse. Stacked bar graphs **(A, B)** show the respective proportions of SP and DN CD90^+^ T cells in each mouse strain. Error bars in graphs show SD. **(B)** Representative flow cytometry plots of CD4 and CD8 expression in gated CD90^+^ T cells. **(C)** Bar graphs show the percentage and absolute number of B220^+^ DN CD90^+^ T cells from each mouse strain. Data are expressed as mean (SD) of 6-8 mice per strain. **(D)** Kinetics of B220^+^ DN CD90^+^ T cell accumulation in LNs of B6/ *lpr* (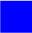), B6/ *lpr* -*p2x7*KO (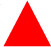), and MRL/ *lpr* (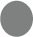) mice measured by flow cytometry. Data are expressed as mean (SD) of 6-7 mice per time point. Error bars in graphs show SD. Asterisks above horizontal lines indicate statistically significant differences assessed with unpaired two-tailed *t*-test. *p ≤ 0.05, **p ≤ 0.01, ***p ≤ 0.001, ****p ≤ 0.0001 and ns, not significant.

T cells that accumulate in MRL/*lpr* mice mainly express the B220^+^ DN phenotype (29). We thus immunophenotyped B6/*lpr*-*p2x7*KO splenocytes (**Figure 2**) and LN cells (data not shown) to characterize and quantify the T-cell subpopulations. B6/*lpr*-*p2x7*KO mice showed significantly higher absolute numbers of CD4^+^ and CD8^+^ T cells and DN T cells than the parental strains (**Figure 2A**). However, the percentage of CD4^+^ T cells among all T cells was unchanged in B6/*lpr*-*p2x7*KO mice, whereas it was significantly lower for CD8^+^ T cells and higher for DN T cells (**Figure 2B**). Moreover, DN T cells expressed the transmembrane phosphatase B220 (**Figure 2C**) and their numbers were markedly higher in B6/*lpr*-*p2x7*KO females than males (data not shown). In sharp contrast to B6/*lpr*-*p2x7*KO mice, < 3% B220^+^ DN T cells were found in age-matched B6 and B6-*p2x7*KO mice (**Figure 2C**).

#### Kinetics of B220^+^ DN T-cell accumulation in B6/*lpr*-*p2x7*KO mice

Macroscopically, adenopathy developed later in the B6/*lpr*-*p2x7*KO than MRL/*lpr* mice. The diameter of the cervical LN was 15.37 mm (SD: 5.98, n = 14) in MRL/*lpr* and B6/*lpr*-*p2x7*KO mice at 12 and 16 weeks of age, respectively, suggesting distinct kinetics of lymphoaccumulation between the two strains. Thus, we monitored the accumulation of B220^+^ DN T cells in the LNs over time. They represented 17% of total T cells in 12-week-old B6/*lpr* and B6/*lpr*-*p2x7*KO mice and nearly 45% in age-matched MRL/*lpr* mice. The percentage of B220^+^ DN T cells continued to increase well beyond 12 weeks of age for the B6/*lpr*-*p2x7*KO but not B6/*lpr* mice, reaching that of MRL/*lpr* mice at 30 weeks of age (**Figure 2D**). In summary, although B6/*lpr* mice generated B220^+^ DN T cells with aging, only B6/*lpr*-*p2x7*KO mice produced the high numbers found in MRL/*lpr* mice. Overall, our data strongly suggest that the *fas* and *p2x7* mutations synergize to strongly amplify the mild lymphoproliferative syndrome of B6/*lpr* mice.

### CD8 SP origin of B220^+^ DN T lymphocytes in B6/*lpr*-*p2x7*KO mice

**Figure 3:**
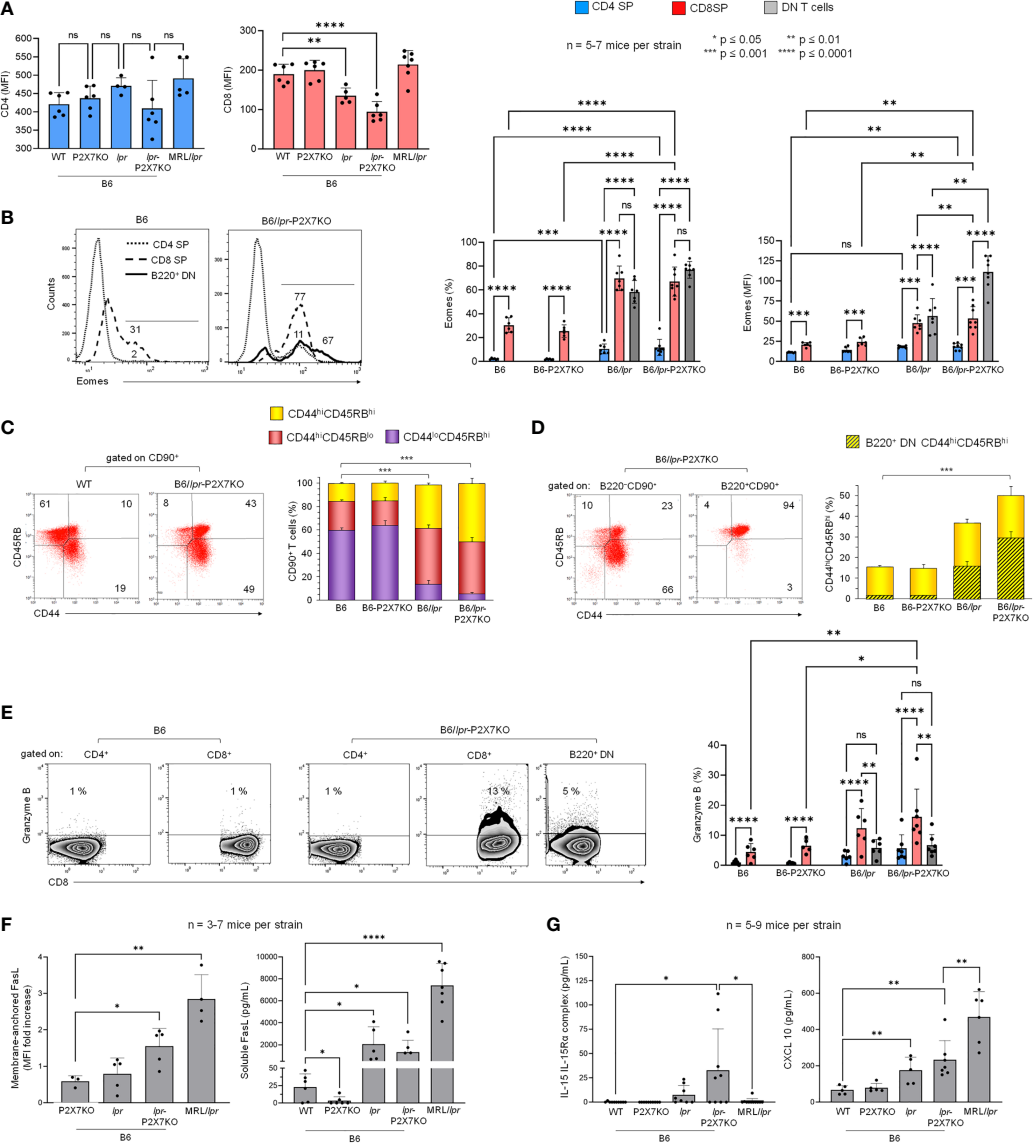
Cellular origin and activation state of B220^+^ DN T lymphocytes that accumulate in B6/*lpr*- *p2x7*KO mice. Spleen cells from 7- to 8-month-old female B6, B6-*p2x7*KO, B6/ *lpr* and B6/ *lpr* -*p2x7*KO and 4-month-old female MRL/ *lpr* mice were stained with either fluorescent mAbs against the phenotypic and functional markers CD90, B220, CD4, CD8, CD44, CD45RB, FasL, Eomes, and Granzyme B or isotype controls and analyzed by flow cytometry. **(A)** Bar graphs show the mean (SD) of median fluorescent intensity (MFI) values for CD4 and CD8 expression on SP CD90^+^ T cells from each mouse strain (n= 5-7 mice per strain). **(B)** Representative flow cytometry histograms of Eomes intracellular staining gated on CD4 SP, CD8 SP and B220^+^ DN CD90^+^ T cells. Bar graphs show the percentage of Eomes^+^ cells (left) and Eomes MFI (right) in gated CD4^+^ (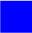), CD8^+^ (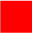), and B220^+^ DN (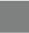) CD90^+^ T cells from each mouse strain. Data are expressed as mean (SD) of 5-7 mice per strain. **(C, D)** Representative flow cytometry plots of CD44/CD45RB staining. CD44 versus CD45RB expression allowed to determine the percentages of naïve (CD44^lo^CD45RB^hi^) and effector/memory (either CD44^hi^CD45RB^lo^ or CD44^hi^CD45RB^hi^) cells in whole CD90^+^ T cells **(C)** and in B220^−^ SP (either CD4 or CD8) and B220^+^ DN CD90^+^ T cells **(D)**. Stacked bar graphs show **(C)** the respective proportions of naïve (CD44^lo^CD45RB^hi^, 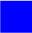) and effector/memory (either 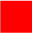
CD44^hi^CD45RB^lo^ or 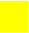
CD44^hi^CD45RB^hi^) cells in CD90^+^ T cells and **(D)** the proportion of B220^+^ DN cells (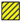) in CD44^hi^CD45RB^hi^ effector/memory CD90^+^ T cells (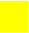) from each mouse strain. Data are expressed as mean (SD) of 5-7 mice per strain. **(E)** Representative flow cytometry dot plots of Granzyme B/CD8 staining in gated CD4 SP, CD8 SP and B220^+^ DN CD90^+^ T cells. Bar graphs show the percentage of Granzyme B^+^ cells in gated CD4^+^ (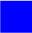), CD8^+^ (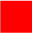), and B220^+^ DN (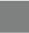) CD90^+^ T cells from each mouse strain. Data are expressed as mean (SD) of 5-7 mice per strain. **(F, G)** Membrane-anchored FasL (**F**, left) on CD90^+^ T cells from each mouse strain. Data are shown as mean fold increase (SD, n = 3-7 mice per strain) in FasL MFI on CD90^+^ T cells relative to staining with an isotype control mAb. Serum levels of soluble FasL (**F**, right), IL-15/IL-15R complex (**G,** left), and CXCL10 (**G,** right) measured by ELISA in the sera of 6- to 7-month-old female B6, B6-*p2x7*KO, B6/ *lpr* , and B6/ *lpr* -*p2x7*KO mice and 4-month-old female MRL/ *lpr* mice Data are shown as mean (SD) of 5-7 mice per strain. Each dot in the bar graphs represents an individual mouse. Error bars in graphs show SD. Asterisks above horizontal lines indicate statistically significant differences assessed with unpaired two-tailed *t*-test in **(A, C, D, F, G)** and two-way ANOVA with a Tukey *post hoc* test in **(B, E)**. *p ≤ 0.05, **p ≤ 0.01, ***p ≤ 0.001, ****p ≤ 0.0001 and ns, not significant.

As the increased percentage of DN T cells in B6/*lpr*-*p2x7*KO spleens was counterbalanced by the decreased percentage of CD8^+^ but not CD4^+^ T cells (**Figure 2B**), we examined whether B6/*lpr*-*p2x7*KO DN T cells originate from CD8 SP T cells by quantifying the levels of membrane-anchored CD8 and the transcription factor eomesodermin (Eomes). Eomes plays a central role in CD8^+^ T-cell homeostasis and function (30) and Th1 lineage commitment (31). CD8, but not CD4, median fluorescent intensity (MFI) values were markedly lower in B6/*lpr*-*p2x7*KO T cells and also, albeit a lesser extent, in B6/*lpr* T cells than in B6 and B6-*p2x7*KO T cells. CD8 MFI values were unchanged in MRL/*lpr* T cells (**Figure 3A**). The percentage of Eomes^+^ CD4^+^ T cells and, to a lesser extent, their Eomes MFI values were significantly higher in B6/*lpr* and B6/*lpr*-*p2x7*KO mice than in B6 and B6-*p2x7*KO mice (**Figure 3B**), indicating higher numbers of Th1 cells in both *lpr* strains. Similarly, the CD8^+^ T-cell population showed a 40% higher abundance of Eomes^+^ cells in *lpr* than non-*lpr* mice, along with significantly higher Eomes MFI values (**Figure 3B**), suggesting that *lpr* CD8^+^ T cells are more highly activated. Importantly, 60 to 75% of pathogenic B220^+^ DN T cells from *lpr* mouse strains expressed Eomes, for which the MFI values were similar to those of CD8^+^ T cells in B6/*lpr* mice and two-fold higher than those of CD8^low^ T cells in B6/*lpr*-*p2x7*KO mice (**Figure 3B**).

We recently reported that effector/memory CD8^+^ and CD4^+^ T-cell subpopulations from B6 mice are mainly of the CD44^hi^CD45RB^hi^ and CD44^hi^CD45RB^lo^ phenotype, respectively (27). Therefore, we analyzed CD44 and CD45RB expression on T cells from B6/*lpr*-*p2x7*KO mice and the parental strains. Approximately 40% of splenic T cells from B6 and B6-*p2x7*KO mice showed the CD44^hi^CD45RB^hi^ or CD44^hi^CD45RB^lo^ effector/memory phenotype (Figure 3C), whereas the value was 70% to 90% in B6/*lpr* and B6/*lpr*-*p2x7*KO mice (**Figure 3C**). Similarly to CD8^+^ T cells, almost all B220^+^ DN T cells showed a CD44^hi^CD45RB^hi^ effector/memory phenotype (**Figure 3D**). Overall, our data suggest that effector/memory Eomes^+^ CD44^hi^CD45RB^hi^ CD8^+^ T cells from B6/*lpr*-*p2x7*KO mice downregulate the expression of membrane-anchored CD8 and become pathogenic Eomes^high^ B220^+^ DN T cells.

### High numbers of granzyme B^+^ CD8^+^ and FasL^+^ DN T cells in B6/*lpr*-*p2x7*KO mice

We investigated the cytotoxic potential of the B6/*lpr*-*p2x7*KO T-cell subpopulations by quantifying their levels of granzyme B (GzmB) and membrane-anchored FasL. GzmB^+^ cell numbers were significantly higher among CD8^+^ T cells than among CD4^+^ and pathogenic B220^+^ DN T cells, irrespective of the mouse strain studied (**Figure 3E**). Membrane-anchored FasL was overexpressed on T cells from B6/*lpr*-*p2x7*KO mice but not those from age-matched parental strains (**Figure 3F**). Moreover, soluble FasL, a biomarker of ALPS found to be overexpressed in certain SLE patients (32, 33), was measured at high levels in B6/*lpr*, B6/*lpr*-*p2x7*KO, and MRL/*lpr* sera (**Figure 3F**).

Furthermore, serum levels of IL-15/IL-15Rα complexes, which upregulate CD8^+^ T-cell homeostasis and function (34), were highly elevated in B6/*lpr*-*p2x7*KO mice, with levels 5 to 20 times higher than those in B6/*lpr* and MRL/*lpr* mice, respectively (**Figure 3G**). Single mutant B6-*p2x7*KO and wildtype B6 mice had similar low levels of IL-15/IL-15Rα complexes. Elevated serum levels of the chemokine CXCL10, which regulates the migration of effector CXCR3^+^ CD8^+^ T cells and GzmB production (35), were also found in B6/*lpr*-*p2x7*KO mice, although to a lesser extent than in MRL/*lpr* mice (**Figure 3G**). Overall, our data suggest that CD8 SP and DN T cells might be a source of potent cytotoxic T cells with strong ability to contribute to the severity of the lupus disease in B6/*lpr*-*p2x7*KO mice.

### Massive leukocyte infiltration into various tissues of B6/*lpr*-*p2x7*KO mice

**Figure 4:**
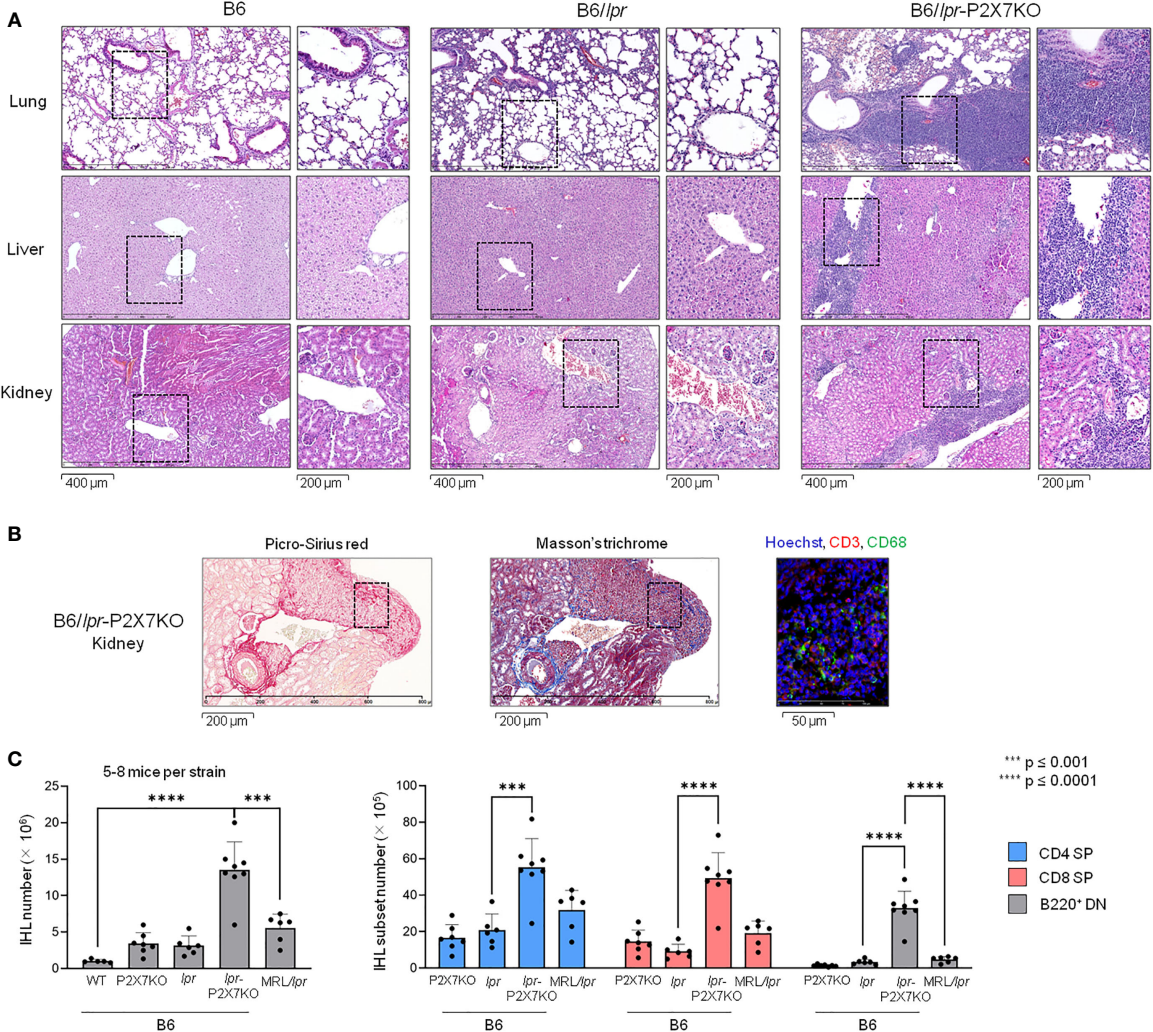
Massive leukocyte infiltration in the liver, lungs, and kidneys of B6/ *lpr* -*p2x7*KO mice. **(A)** Representative photomicrograph (bar scale = 400 μm) of histological examination of mononuclear cell infiltrates in lung, kidney, and liver sections from 6- to 7-month-old female B6, B6/ *lpr* and B6/ *lpr* -*p2x7*KO mice. The corresponding position of the inset is indicated by a black dotted line in the photomicrographs. The inset photomicrograph (bar scale = 200 µm) is shown in the second column of the panel for each mouse strain and tissue. Histological photomicrographs were captured using a digital camera. **(B)** Renal tissue sections from B6/ *lpr* -*p2x7*KO mice were labelled with anti-CD68 and anti-CD3 fluorescent mAbs. Sections were stained with picro-sirius red and Masson’s trichrome to detect renal fibrosis. **(C)** Infiltrating leukocytes isolated from individual livers of 7- to 8-month-old female B6/ *lpr* , B6-*p2x7*KO and B6/ *lpr* -*p2x7*KO mice and 4-month-old female MRL/ *lpr* mice were stained with either fluorescent mAbs against the phenotypic markers CD90, B220, CD4, and CD8 or isotype controls and analyzed by flow cytometry. Bar graphs show the mean percentage (SD, n = 5-8 livers per strain) of total CD90^+^ T cells (IHL, left panel), as well as CD4^+^ (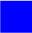), CD8^+^ (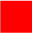) and B220^+^ DN (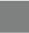) CD90^+^ T-cell subsets. Each dot in the bar graphs represents an individual mouse. Error bars in graphs show SD. Asterisks above horizontal lines indicate statistically significant differences assessed with unpaired two-tailed *t*-test. ***p ≤ 0.001, ****p ≤ 0.0001.

Pulmonary disease and nephritis are an important cause of morbidity and mortality in SLE patients. Histological analysis of lung and kidney sections from B6/*lpr*-*p2x7*KO mice showed an abnormal structure, with widespread peribronchiolar, periglomerular, and perivascular mononuclear cell infiltrates (**Figure 4A**) consisting predominantly of CD68^+^ macrophages and T cells in renal tissue (**Figure 4B**). Leukocyte infiltrates were never found in B6/*lpr* and wildtype B6 tissues. Hepatomegaly and/or liver disease can occur in certain SLE patients. Interestingly, B6/*lpr*-*p2x7*KO mice, but not the parental strains, exhibited increased liver weights (**Table 1**), consistently associated with the presence of perivascular and parenchymal mononuclear cell infiltrates (**Figure 4A**). Tissue injury in B6/*lpr*-*p2x7*KO mice was confirmed by large areas of renal tissue fibrosis (**Figure 4B**) and elevated serum levels of urea (17.8 mM), ASAT (899.5 U/L), and LDH (4352 U/L). Overall, our data suggest that early mortality in B6/*lpr*-*p2x7*KO mice is primarily due to renal failure.

### B220^+^ DN T cells massively infiltrate B6/*lpr*-*p2x7*KO livers

We isolated and immunophenotyped intrahepatic lymphocytes (IHLs). CD4^+^ and CD8^+^ IHL counts were significantly higher in B6/*lpr*-*p2x7*KO mice than in the parental strains and even MRL/*lpr* mice (**Figure 4C**). Similarly, B220^+^ DN T-cell numbers were 7- and 10-fold higher among IHLs from B6/*lpr*-*p2x7*KO than MRL/*lpr* and B6/*lpr* mice, respectively.

### Memory T-cell subsets predominate within the T-cell population of B6/*lpr*-*p2x7*KO mice

**Figure 5:**
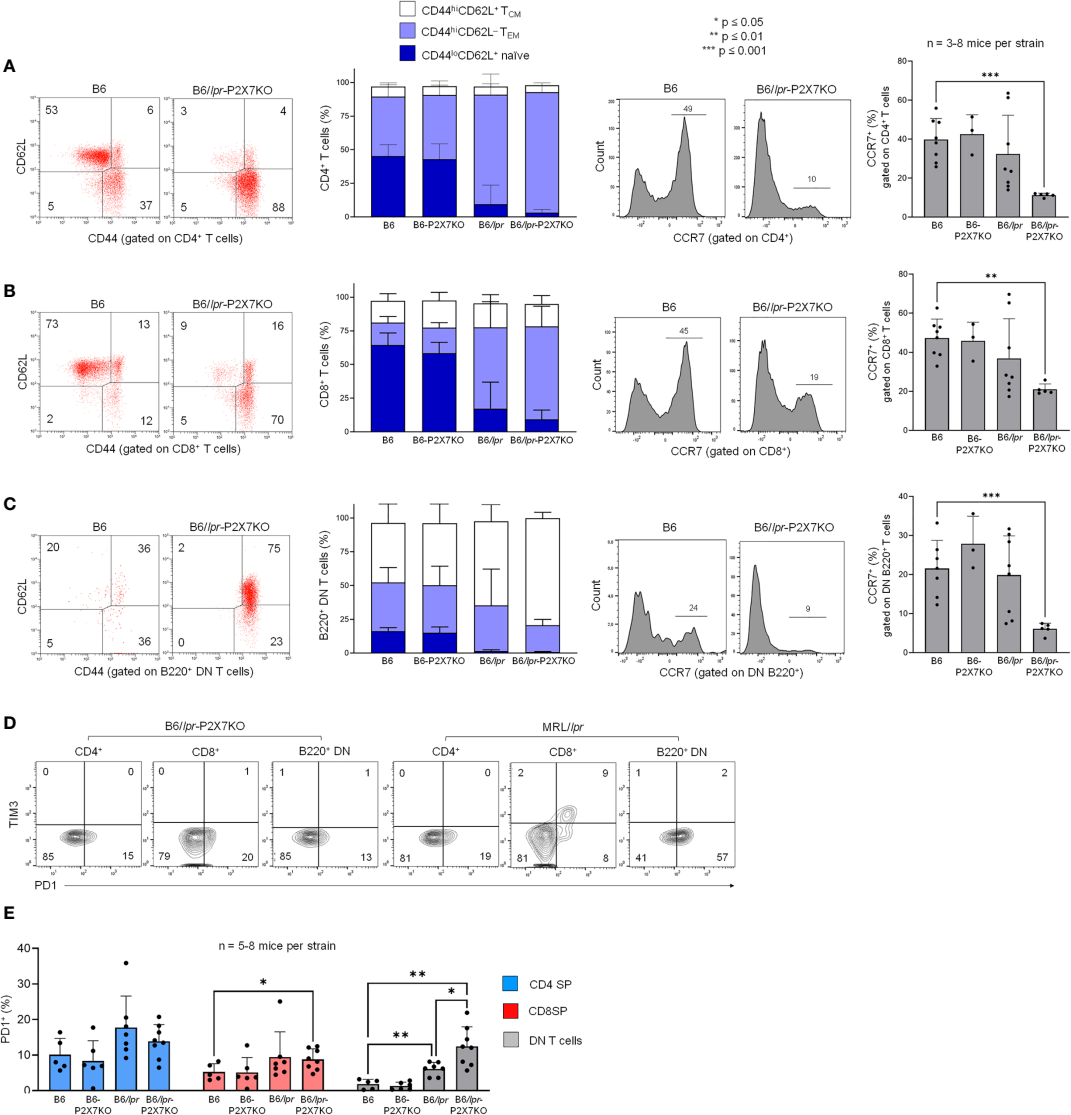
T cell subsets in B6*/ *lpr**-*p2x7*KO express predominantly central memory and effector memory phenotype but not PD-1^+^Tim-3^+^ exhausted phenotype. Spleen cells from 7- to 8-month-old female wild-type B6, B6-*p2x7*KO, B6/ *lpr* and B6/ *lpr* -*p2x7*KO mice **(A–E)** and 4-month-old female MRL/ *lpr* mice **(D)** were stained with either fluorescent mAbs against phenotypic markers CD90, B220, CD4, CD8 CD44, CD62L, CCR7, PD-1, Tim-3 or isotype controls and analyzed by flow cytometry. **(A–C)** Representative flow cytometry dot plots of CD44/CD62L staining. CD44 versus CD62L expression allowed to quantify the percentages of CD44^lo^CD62L^+^ naïve (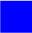), CD44^hi^CD62L^−^ effector memory (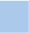, T_EM_) and CD44^hi^CD62L^+^ central memory (□, T_CM_) cells on gated CD4 SP **(A)**, CD8 SP **(B)** or B220^+^ DN **(C)** CD90^+^ T cells from each mouse strain. Stacked bar graphs (**A–C**, second panel) show the respective proportions of naive, T_EM_ and T_CM_ cells in CD4^+^, CD8^+^ and B220^+^ DN CD90^+^ T cells from each mouse strain. (**A–C**, third panel) Representative flow cytometry histograms of CCR7 staining gated on CD4 SP **(A)**, CD8 SP **(B)** or B220^+^ DN **(C)** CD90^+^ T cells from each mouse strain. (**A–C**, fourth panel) Bar graphs show the mean percentage (SD, n = 3-8 mice per strain) of CCR7-expressing CD4 SP, CD8 SP and B220^+^ DN CD90^+^ T cells from each mouse strain. **(D)** Quantification of PD-1^+^Tim-3^+^ exhausted T cells by flow cytometry. Representative dot plots of PD-1/Tim-3 staining gated on CD4 SP, CD8 SP and B220^+^ DN CD90^+^ T cells. **(E)** Bar graphs show the mean percentage (SD, n = 5-8 mice per strain) of PD-1^+^ cells in gated CD4 SP (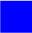), CD8 SP (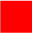) or B220^+^ DN (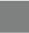) CD90^+^ T cells from each mouse strains. Each dot in the bar graphs represents an individual mouse. Error bars in graphs show SD. Asterisks above horizontal lines indicate statistically significant differences assessed with unpaired two-tailed *t*-test. *p ≤ 0.05, **p ≤ 0.01, ***p ≤ 0.001.

The high levels of IL-15/IL-15Rα complexes in B6/*lpr*-*p2x7*KO mice led us to assess the predominance of effector memory (TEM) and central memory (TCM) T-cell subsets within the SP and DN T-cell subsets from B6/*lpr*-*p2x7*KO mice, as IL-15/IL-15Rα complexes regulate their expansion. B6/*lpr*-*p2x7*KO and B6/*lpr* mice exhibited a lower percentage of CD44^lo^CD62L^+^ naïve CD4^+^ and CD8^+^ T cells, but a higher percentage of CD44^hi^CD62L^−^CCR7^−^ TEM and CD44^hi^CD62L^+^CCR7^+^ TCM subsets than B6 and B6-*p2x7*KO mice (**Figures 5A, B**). Interestingly, most pathogenic B220^+^ DN T cells from B6/*lpr*-*p2x7*KO and B6/*lpr* mice had an unusual CCR7-negative CD44^hi^CD62L^+^ phenotype (**Figure 5C**), along with a CD45RB^hi^ phenotype (**Figure 3C**), reminiscent of the human EMRA phenotype [CD45RA^hi^CD44^hi^CCR7^−^ (36, 37)] of chronically activated CD8^+^ T cells. Overall, our data suggest that SP and DN T-cell subsets from *lpr* mice, especially B6/*lpr*-*p2x7*KO mice, are chronically activated, as observed under conditions of severe autoimmunity. Therefore, we evaluated whether such chronic activation drives T cells to an exhausted fate. However, PD-1^+^Tim-3^+^ T cells were completely absent from B6/*lpr*-*p2x7*KO mice (**Figures 5D, E**), indicating that the accumulating T cells in B6/*lpr*-*p2x7*KO mice do not show phenotypic features of an exhausted state.

### Normal percentage of DP and SP thymocytes but an elevated percentage of pre-B cells in B6/*lpr*-*p2x7*KO mice

Absolute numbers of T cells, but not B cells, were strongly elevated in B6/*lpr*-*p2x7*KO mice (**Figure 1**). We therefore determined whether the combined mutations of *fas* and *p2x7* genes affect T cell maturation. The percentage of DN, CD4^+^CD8^+^, CD4 SP, and CD8 SP thymocyte subsets was similar in B6/*lpr*-*p2x7*KO and B6 mice. Similarly, DN thymocyte subsets (DN1 to DN4) defined on the basis of CD44 and CD25 expression were found in similar proportions in B6/*lpr*-*p2x7*KO and B6 mice (**Figures S3A, C**). Unexpectedly, we found a strong reduction in the percentage of IgM^+^IgD^+^ transitional B cells and a compensatory increase in the percentage of IgM^−^IgD^−^ pre-B cells (**Figure S3B, D**) in B6/*lpr*-*p2x7*KO bone marrow.

### A dysregulated cytokine network and high levels of autoantibodies in B6/*lpr*-*p2x7*KO mice

**Figure 6:**
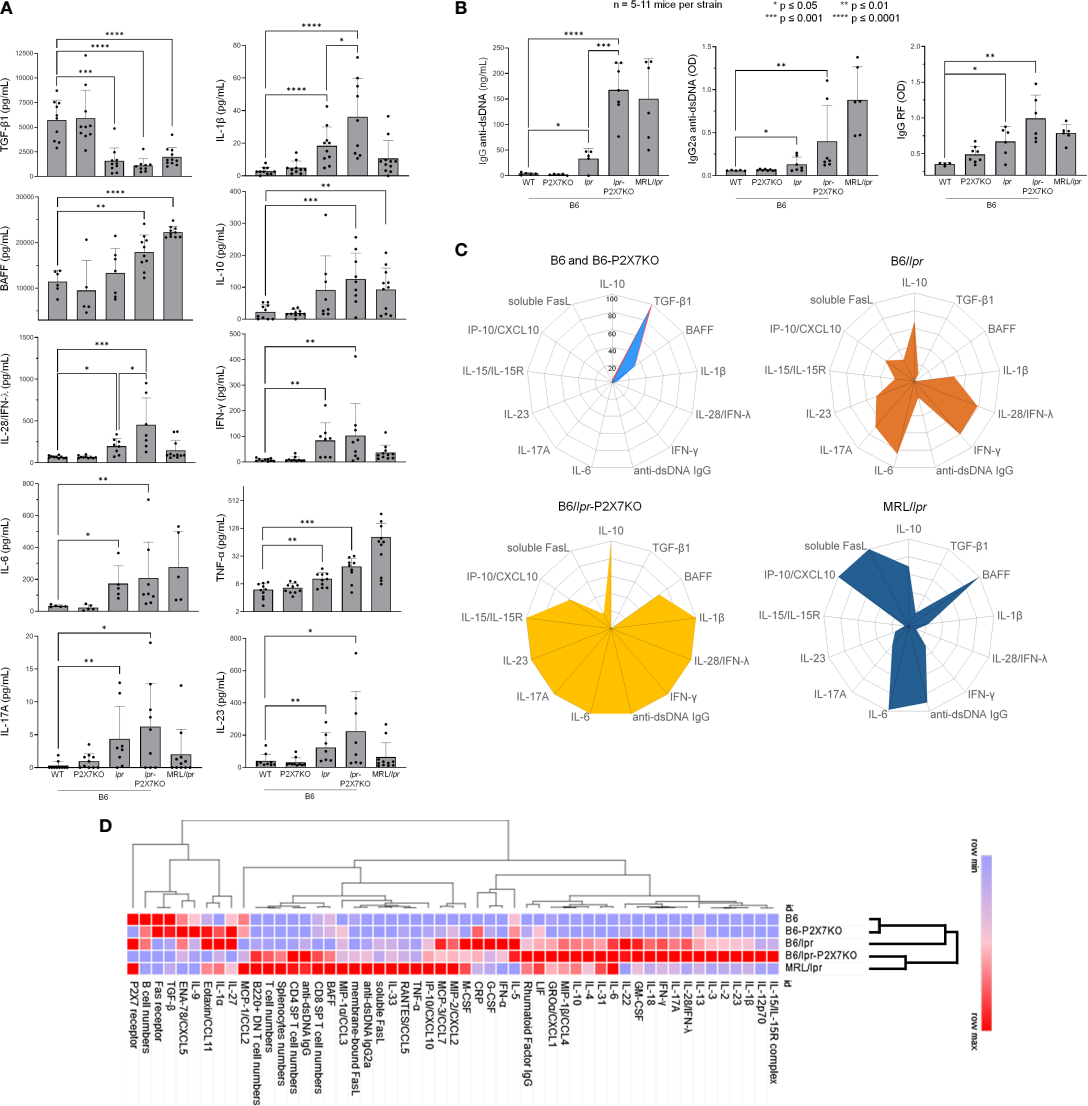
Cytokine and autoantibody levels in sera from B6*/ *lpr**-*p2x7*KO mice and parental strains as well as hierarchical clustering of autoimmune features. Bar graphs show serum levels of **(A)** the cytokines TGF-β1, IL-10, BAFF, IFN-γ, IL-28/IFN-λ, IL-1β, IL-6, TNF-α, IL-17A, and IL-23, as well as **(B)** total IgG and IgG2a anti-dsDNA and IgG RF autoantibodies in 6- to 7-month-old female and male wildtype B6, single-mutant B6-*p2x7*KO, and B6/ *lpr* mice and double-mutant B6/ *lpr* -*p2x7*KO mice, as well as 4-month-old female MRL/ *lpr* mice. Data are expressed as mean (SD) of 5-11 mice per strain, and each dot in the bar graphs represents an individual mouse. Error bars in graphs show SD. Asterisks above horizontal lines indicate statistically significant differences assessed with unpaired two-tailed *t*-test. *p ≤ 0.05, **p ≤ 0.01, ***p ≤ 0.001 ****p ≤ 0.0001. **(C)** Radar plot profiles of the cytokine and anti-dsDNA autoantibody levels in B6 and B6-*p2x7*KO (upper panel, left), B6/ *lpr* (upper panel, right), B6/ *lpr* -*p2x7*KO (lower panel, left), and MRL/ *lpr* (lower panel, right) mice. Using min-max normalization, we rescaled data values of every feature between 0 and 1 *via* the formula x’ = (x – min(x))/(max(x) – min(x)) where min(x) and max(x) are the minimum and the maximum values of the feature, respectively. **(D)** Heat map and hierarchical clustering of cellular and serological features from B6, B6-*p2x7*KO, B6/ *lpr* , B6/ *lpr* -*p2x7*KO, and MRL/ *lpr* mice. For each feature, measured data were normalized (min-max normalization) and loaded in the data-processing software Morpheus (Broad Institute). The color gradient represented the lowest (blue) and highest (red) value found among the five mice strains. Hierarchical clustering was applied using one minus Pearson correlation to assess whether clinical and biological parameters from B6/ *lpr* -*p2x7*KO cluster with those of B6/ *lpr* and/or MRL/ *lpr* mice.

Dysregulation of the cytokine network plays a key role in the pathogenesis of ALPS and SLE (1, 29). We therefore measured serum cytokine levels in B6/*lpr*-*p2x7*KO mice. Serological analyses (**Figure 6**) were mostly performed on the same individual mice that those used in lymphocyte immunophenotyping experiments (**Figures 3**, **5**). Wildtype B6 and single-mutant B6-*p2x7*KO mice expressed high levels of TGF-β1 and low levels of IL-10 and proinflammatory cytokines. By contrast, sera from B6/*lpr*-*p2x7*KO mice and, to a lesser extent, B6/*lpr* mice contained low levels of TGF-β1 and high levels of IL-10. Moreover, B6/*lpr*-*p2x7*KO sera contained high levels of the proinflammatory cytokines IFN-γ, IL-28/IFN-λ, IL-1β, IL-6, TNF-α, IL-17A, and IL-23, of which the levels were similar or even higher (except for TNF-α) than in MRL/*lpr* sera (**Figure 6A**). The **Table S1** presents cytokines, such as IL-12p70 or IL-18, whose levels were found higher in B6/*lpr*-*p2x7*KO sera than parental strains or MRL/*lpr* sera, but this trend of overexpression failed to reach statistical significance due to high inter-individual variability. We also present in this table the cytokines whose levels remained unaltered in B6/*lpr*-*p2x7*KO mice.

As TGF-β1 plays a central role in the generation, expansion, and survival of Foxp3^+^ regulatory CD4^+^ T cells (Tregs), we quantified Tregs in B6/*lpr* and B6/*lpr*-*p2x7*KO mice. Unexpectedly, B6/*lpr*-*p2x7*KO mice showed significantly higher numbers of Foxp3^+^ Tregs than the parental strains (**Figure S4**), suggesting that Fas and P2X7 may synergistically control the pool size of Tregs in the periphery.

Anti-dsDNA autoantibodies are closely associated with manifestation of end-organ damage in patients and mouse models. RF is often associated with rheumatoid arthritis and Sjogren’s syndrome (38). As expected, we measured pathological levels of IgG, in particular complement-fixing IgG2a, dsDNA-reactive antibodies, and RF IgG in the sera of B6/*lpr* but not B6-*p2x7*KO mice (**Figure 6B**). Importantly, serum levels of IgG (**Figure 6B**, left panel), IgG2a, (**Figure 6B**, middle panel), and RF autoantibodies (**Figure 6B**, right panel) in B6/*lpr*-*p2x7*KO mice were significantly higher than in B6/*lpr* mice. Moreover, numerous studies have emphasized the central role of the cytokines BAFF, IL-6, and IL-10 in the production of autoantibodies, as well as the role of IFN-γ in isotype switching to nephritogenic complement-fixing IgG autoantibodies. Interestingly, in addition to IL-6, IL-10, and IFN-γ (see above), we found overexpression of BAFF in the sera of B6/*lpr*-*p2x7*KO mice (**Figure 6A**), suggesting that the dysregulated production of these cytokines is responsible for the expression of SLE-associated serological biomarkers in B6/*lpr*-*p2x7*KO mice.

Finally, to have a more global perspective of autoimmune dysregulations developed by the B6/*lpr*-*p2x7*KO mouse strain, cellular and serological data were normalized and presented as radar plots and heat map matrix (**Figures 6C, D**). Hierarchical clustering was also performed using Morpheus software (Broad institute). Importantly, while B6/*lpr*-*p2x7*KO and MRL/*lpr* mice were clustered together, B6-*p2x7*KO mice were clustered with normal B6 mice, highlighting the severity of the disease developed by the novel B6/*lpr*-*p2x7*KO mouse strain. Thus, B6/*lpr*-*p2x7*KO and MRL/*lpr* mice shared similar dysregulations in the proportion of the normal and pathogenic T-cell subpopulations (**Figure 6D**). However, at the serological level, B6/*lpr*-*p2x7*KO mice displayed a unique cytokine profile which combines some of the cytokine dysregulations found in B6/*lpr* and/or MRL/*lpr* mice and some found in B6/*lpr*-*p2x7*KO mice only (**Figures 6C, D**). In agreement with their mild auto-immune phenotype, B6/*lpr* mice were clustered between the normal (B6 and B6-*p2x7*KO) and the severely affected (B6/*lpr*-*p2x7*KO and MRL/*lpr*) group (**Figure 6D**).

Overall, our data reveal that combined Fas and P2X7 deficiency massively amplified the mild autoimmune phenotype of B6/*lpr* mice at both the cellular and serological levels.

## Discussion

ALPS is characterized by disrupted peripheral lymphocyte homeostasis, leading to splenomegaly, lymphadenopathy, and hepatomegaly. Although ALPS is frequently due to germline mutations of the *FAS, FASLG,* or *CASP10* apoptosis gene, the genetic basis of the disease is unknown for a significant number of patients (1). Most ALPS patients present autoimmune cytopenia. As in SLE, certain ALPS patients with mostly unidentified genetic defects develop skin rashes, pulmonary disease, glomerulonephritis, hepatitis, and arthritis (39). SLE is a multifactorial autoimmune disease characterized by the hyperactivation of immune cells, the production of autoantibodies, and immune complex-mediated inflammatory damage in multiple organs. The genetic factors that contribute to predisposition to the disease and its severity consist of multiple susceptibility alleles, each with only a small individual contribution. Although MRL/*lpr* and B6/*lpr* mouse models have allowed the discovery of key mechanisms of the disease (2), additional genes of pathological importance are yet to be discovered.

The P2X7/ATP pathway plays a major role in the development of inflammatory and autoimmune diseases (14, 16). Thus, elevated P2X7 expression on Th1 and Th17 cells from SLE patients has been shown to be related to disease exacerbation (20), whereas P2X7 deletion in follicular helper T cells led to their aberrant expansion and the generation of self-reactive antibodies in pristane-induced lupus mice (25). However, we did not observe an expansion of the Bcl-6-expressing effector/memory CD4^+^ T-cell subset in B6/*lpr*-*p2x7*KO mice (data not shown). These previous reports, showing either a pathogenic or a protective role of P2X7 in the onset and progression of SLE, highlight the need to pursue studies on how P2X7 regulates T-cell effector functions under normal and pathological conditions. Previously, we showed that P2X7 expression and ATP-induced cellular functions in normal CD4^+^ (conventional and regulatory) and CD8^+^ T cells depend on their stage of activation and differentiation instead of P2X7 levels (26, 27). We previously showed a marked downregulation of P2X7 membrane expression and ATP sensitivity in pathogenic CD4^–^CD8^–^ T cells from MRL/*lpr* and B6/*lpr* mice (28), suggesting that P2X7 deficiency could amplify lymphoaccumulation and autoimmunity in the Fas-deficient mouse strain. Therefore, we generated double-mutant B6/*lpr*-*p2x7*KO mice. We chose the B6 genetic background because B6/*lpr* mice develop milder disease than MRL/*lpr* mice, making it easier to detect potential phenotypic changes. Importantly, B6/*lpr*-*p2x7*KO mice showed an unexpected high early death rate, indicating that they develop a rapid and/or severe form of the disease. Thus, mean survival for B6/*lpr*-*p2x7*KO mice was 20 weeks for both males and females versus 60 (females) to 73 (males) weeks for B6/*lpr* mice [**Table 2** and (10)]. These data on mortality rates by sex suggest an absence of sexual dimorphism in B6/*lpr*-*p2x7*KO mice, although females showed significantly higher lymphoaccumulation than males. Lymphadenopathy and splenomegaly in B6/*lpr*-*p2x7*KO mice, which peaks at approximately 7 to 8 months of age, were due to the massive accumulation of FasL^hi^B220^+^ DN T cells expressing the EomeshiCD44^hi^CD45RB^hi^ effector/memory phenotype. Moreover, we found B220^+^ DN T cells to mostly originate from Eomes^+^ CD8^+^ T cells at the CD44^hi^CD45RB^hi^ effector/memory stage, which display a very low sensitivity to both P2X7 (27) and Fas receptor (40). Importantly, Eomes-deficient B6/*lpr* mice show reduced B220^+^ DN T cell numbers (30), confirming the CD8 origin of the DN subset. Moreover, B6/*lpr*-*p2x7*KO sera contained high levels of IFN-γ, of which the important sources of production are effector/memory CD8^+^ T cells. Overall, our data suggest that P2X7 is physiologically involved in the homeostasis of effector/memory CD8^+^ T cells in synergy with the Fas death receptor. Finally, the high levels of CD45RB on B220^+^ DN T cells from B6/*lpr*-*p2x7*KO mice, which is reminiscent of the human EMRA phenotype [CD44^hi^CD45RA^hi^CCR7^−^ (36, 37)], suggest that they are in a chronically activated state. In addition, PD1^+^Tim-3^+^ T cells were entirely absent from B6/*lpr*-*p2x7*KO mice, indicating that accumulating T cells do not reach an exhausted state.

Importantly, the limited lupus-like syndrome of B6/*lpr* mice was greatly amplified in B6/*lpr*-*p2x7*KO mice, as shown by the high levels of complement-fixing IgG2a anti-dsDNA autoantibodies (**Table 2**), which are major nephritogenic autoantibodies in MRL/*lpr* mice (41), and the dysregulated cytokine network. The key synergistic role of the Fas and P2X7 deficiency in the severity of lupus disease is emphasized by the low anti-dsDNA antibody levels in Bcl-3 (42) and NF-κB2 (43) deficient B6/*lpr* mice, although they display high B220^+^ DN T-cell numbers. In B6/*lpr*-*p2x7*KO sera, we found high levels of IFN-γ, which is involved in isotype switching to complement-fixing IgG2a, as well as BAFF, IL-10, IL-6, and IL-17A. The latter cytokines are instrumental in driving autoantibody production in lupus through their ability to control B-cell maturation, survival, and differentiation into antibody-secreting plasma cells. Therefore, overproduced long-lived plasma cells could be involved in the decreased percentage of transitional B cells found in B6/*lpr*-*p2x7*KO bone marrow. IL-6 is also essential for the generation and maintenance of IL-17A-producing Th17 cells, which are involved in ALPS and SLE by protecting T cells from Fas-induced cell death (44). Indeed, lower numbers of IL-17A-producing Th17 cells in IL-23R-deficient B6/*lpr* mice resulted in reduced anti-DNA antibody levels and lupus nephritis (45). Of note, DN T cells are an important source of IL-17A in SLE patients (4) and MRL/*lpr* mice (46), and therefore most probably also in B6/*lpr*-*p2x7*KO mice. As in various autoimmune diseases (47), IL-15/IL-15Rα complexes were found at high levels in B6/*lpr*-*p2x7*KO sera. Such overexpression could amplify lymphoaccumulation, as the IL-15/IL-15Rα complex is an anti-apoptotic cytokine (48), in addition to its ability to regulate CD8^+^ T-cell expansion and cytotoxicity (34). Sera of B6/*lpr*-*p2x7*KO mice also contained high levels of the type III interferons IL-28/IFN-λ, for which the role in SLE pathogenesis has been recently highlighted (49). Importantly, B6/*lpr*-*p2x7*KO mice had low levels of serum TGF-β1, which is a predisposing factor to autoimmunity, as TGF-β1 plays a pivotal role in maintaining peripheral immune tolerance by promoting Foxp3^+^ Tregs (50). Moreover, the overexpression of IL-6 in B6/*lpr*-*p2x7*KO mice could further impair the generation of peripheral Foxp3^+^ Tregs, as high IL-6 levels can favor the conversion of Treg cells into pathogenic IL-17A-secreting Th17 cells (51). However, since Tregs from WT B6 mice express high levels of P2X7 membrane expression and sensitivity to ATP (26, 27), the higher number of Tregs that we observed in B6/*lpr*-*p2x7*KO spleen compared to B6/*lpr* spleen is likely due to their loss of sensitivity to ATP-mediated cell death via P2X7. Our results suggest that P2X7 plays a role in the homeostasis of peripheral Foxp3^+^ Tregs by preventing their tissue accumulation through their high sensitivity to ATP-induced cell death. Besides, P2X7 could negatively regulate the activation/differentiation of Tregs by a mechanism independent of the TGF-β1 pathway that needs to be further investigated.

In summary, we show that B6/*lpr*-*p2x7*KO mice express key biomarkers of ALPS and severe lupus disease (1, 32, 33). Thus, B6/*lpr*-*p2x7*KO mice have large areas of renal tissue fibrosis, high serum levels of inflammatory cytokines, soluble FasL, anti-dsDNA autoantibodies, and RF but they did not show the severe skin lesions observed in MRL/*lpr* mice. However, the livers of B6/*lpr*-*p2x7*KO mice, but not MRL/*lpr* mice, showed massive T-cell infiltrates, especially B220^+^ DN T cells, suggesting that target organs/tissues differ between the two SLE models. Moreover, B6/*lpr*-*p2x7*KO mice showed a significantly higher percentage and/or absolute number of GzmB^+^ CD8 SP T cells and FasL^+^ B220^+^ DN T cells, which could generate tissue damage, as the *lpr* mutation is leaky. In addition, we found elevated blood levels of urea, ASAT, and LDH. Therefore, alongside invaluable models of ALPS and/or SLE, such as MRL/*lpr* mice, the B6/*lpr*-*p2x7*KO mouse strain is a novel model of T-cell homeostasis dysregulation and lupus development. Importantly, B6/*lpr*-*p2x7*KO mice exhibits a unique autoimmune profile highlighting the Fas and P2X7 synergy. Thus some autoimmune features such as renal infiltrations, IgG dsDNA autoantibodies as well as BAFF and IL-6 cytokines are shared between B6/*lpr*-*p2x7*KO and MRL/*lpr* mice, whereas other features such as liver infiltration and IL-1β, IL-15/IL-15R, IL-17A, IL-23, IL-28/IFN-λ are inherited from B6/*lpr* mice but with a massive amplification due to the absence of P2X7. Therefore, our present study is the first experimental demonstration that P2X7 plays a key protective role against the development of ALPS and systemic autoimmune conditions, in particular SLE. The mTOR signaling pathway, which regulates many cellular processes, could play a key role in the protective function of P2X7. Indeed, the treatment of MRL/*lpr* mice with the immunosuppressant FTY720 led to a significant decrease of the number of DN T cells (52), most likely through the activation of protein phosphatase 2A (PP2A), which negatively regulates the mTOR signaling pathway. The involvement of the mTOR kinase in the acquisition of the DN T cell phenotype is further documented by a report showing that the treatment of SLE patients with mTOR inhibitor sirolimus/rapamycin can significantly reduce IL-17-producing DN T cell numbers (53). In patients with ALPS, rapamycin treatment massively reduced lymphoproliferation and numbers of circulating DN T cells. The mTOR pathway has been identified as a major regulator of the accumulation and aberrant differentiation of Fas-deficient T cells (54). Furthermore, the mTOR signaling pathway is activated by proinflammatory cytokines such as IL-15 (55), whereas it can be repressed by the immunosuppressive cytokine TGF-β1 (56) and after P2X7 activation (57). Therefore, we suggest that the high mTOR activity caused by the Fas deficiency in B6/*lpr* T cells could be increased still further by the loss of P2X7, therefore leading to the massive increase of lymphoaccumulation and aberrant differentiation observed in B6/*lpr*-*p2x7*KO mice correlated with the increase of IL-15 and the decrease of TGF-β1 levels.

## Data availability statement

The raw data supporting the conclusions of this article will be made available by the authors, without undue reservation.

## Ethics statement

The animal study was reviewed and approved by local (CEEA03 and CEEA40) and national (MESRI) ethics committees for research (agreement number: D452346).

## Author contributions

The experiments were conceived and designed by PB, AM, TH-H, and JL. JK and KB contributed critical reagent, resources, and expertise. Experiments were performed by AM, TH-H, JL, MB, HS, and FM-N. The data were processed and analyzed by AM, PB, TH-H, JL, and FM-N and supervised by PB. The manuscript was written by PB and AM and critically reviewed by all authors. All authors contributed to the article and approved the submitted version.

## Funding

This work was funded by grants from the ANR – Agence Nationale de la Recherche (ANR-13-ISV6-0003). HS was the recipient of a National Council for Scientific Research (CNRS) Lebanon fellowship.

## Acknowledgments

The authors are grateful to Dr Hélène Le Buanec (INSERM U976, Paris) for giving us access to her BioPlex MAGPIX multiplex reader instrument. Article processing charges have been paid by UMR-996 INSERM – Paris-Saclay University, Team I" Immunoregulation, Chemokines and Viral Persistence".

## Conflict of interest

The authors declare that the research was conducted in the absence of any commercial or financial relationships that could be construed as a potential conflict of interest.

## Publisher’s note

All claims expressed in this article are solely those of the authors and do not necessarily represent those of their affiliated organizations, or those of the publisher, the editors and the reviewers. Any product that may be evaluated in this article, or claim that may be made by its manufacturer, is not guaranteed or endorsed by the publisher.

## References

[1] Molnar E Radwan N Kovacs G Andrikovics H Henriquez F Zarafov A. Key diagnostic markers for autoimmune lymphoproliferative syndrome with molecular genetic diagnosis. Blood (2020) 136(17):1933–45. doi: 10.1182/blood.2020005486 32599613

[2] Perry D Sang A Yin Y Zheng YY Morel L. Murine models of systemic lupus erythematosus. J BioMed Biotechnol (2011) 2011:271694. doi: 10.1155/2011/271694 21403825 PMC3042628

[3] Rodriguez-Rodriguez N Apostolidis SA Fitzgerald L Meehan BS Corbett AJ Martin-Villa JM. Pro-inflammatory self-reactive tcells are found within murine TCR-αβ(+) CD4(-) CD8(-) PD-1(+) cells. Eur J Immunol (2016) 46(6):1383–91. doi: 10.1002/eji.201546056 PMC4913481 27060346

[4] Li H Adamopoulos IE Moulton VR Stillman IE Herbert Z Moon JJ. Systemic lupus erythematosus favors the generation of IL-17 producing double negative T cells. Nat Commun (2020) 11(1):2859. doi: 10.1038/s41467-020-16636-4 32503973 PMC7275084

[5] Hao Z Duncan GS Seagal J Su YW Hong C Haight J. Fas receptor expression in germinal-center b cells is essential for T and b lymphocyte homeostasis. Immunity (2008) 29(4):615–27. doi: 10.1016/j.immuni.2008.07.016 PMC3470429 18835195

[6] Benihoud K Bonardelle D Bobé P Kiger N. MRL/*lpr* CD4- CD8- and CD8+ T cells, respectively, mediate fas-dependent and perforin cytotoxic pathways. Eur J Immunol (1997) 27(2):415–20. doi: 10.1002/eji.1830270211 9045912

[7] Bonardelle D Benihoud K Kiger N Bobé P. B lymphocytes mediate fas-dependent cytotoxicity in MRL/*lpr* mice. J Leukoc Biol (2005) 78(5):1052–9. doi: 10.1189/jlb.0904536 16204618

[8] Bobé P Bonardelle D Reynes M Godeau F Mahiou J Joulin V. Fas-mediated liver damage in MRL hemopoietic chimeras undergoing *lpr*-mediated graft-versus-host disease. J Immunol (1997) 159(9):4197–204. 9379013

[9] Bonardelle D Bobé P Reynes M Amouroux J Tricottet V Godeau F. Inflammatory arthropathy in MRL hematopoietic chimeras undergoing fas mediated graft-versus-host syndrome. J Rheumatol (2001) 28(5):956–61. 11361222

[10] Izui S Kelley VE Masuda K Yoshida H Roths JB Murphy ED. Induction of various autoantibodies by mutant gene *lpr* in several strains of mice. J Immunol (1984) 133(1):227–33. 6609979

[11] Benihoud K Bonardelle D Soual-Hoebeke E Durand-Gasselin I Emilie D Kiger N. Unusual expression of LINE-1 transposable element in the MRL autoimmune lymphoproliferative syndrome-prone strain. Oncogene (2002) 21(36):5593–600. doi: 10.1038/sj.onc.1205730 12165858

[12] Elliott JI McVey JH Higgins CF. The P2X7 receptor is a candidate product of murine and human lupus susceptibility loci: A hypothesis and comparison of murine allelic products. Arthritis Res Ther (2005) 7(3):R468–75. doi: 10.1186/ar1699 PMC1174943 15899033

[13] Portales-Cervantes L Nino-Moreno P Doniz-Padilla L Baranda-Candido L Garcia-Hernandez M Salgado-Bustamante M. Expression and function of the P2X(7) purinergic receptor in patients with systemic lupus erythematosus and rheumatoid arthritis. Hum Immunol (2010) 71(8):818–25. doi: 10.1016/j.humimm.2010.05.008 20493226

[14] Giuliani AL Sarti AC Falzoni S Di Virgilio F. The P2X7 receptor-Interleukin-1 liaison. Front Pharmacol (2017) 8:123. doi: 10.3389/fphar.2017.00123 28360855 PMC5353276

[15] Le Gall SM Bobé P Reiss K Horiuchi K Niu XD Lundell D. ADAMs 10 and 17 represent differentially regulated components of a general shedding machinery for membrane proteins such as transforming growth factor alpha, l-selectin, and tumor necrosis factor alpha. Mol Biol Cell (2009) 20(6):1785–94. doi: 10.1091/mbc.e08-11-1135 PMC2655247 19158376

[16] Kopp R Krautloher A Ramirez-Fernandez A Nicke A. *p2x7* interactions and signaling - making head or tail of it. Front Mol Neurosci (2019) 12:183. doi: 10.3389/fnmol.2019.00183 31440138 PMC6693442

[17] Atarashi K Nishimura J Shima T Umesaki Y Yamamoto M Onoue M. ATP drives lamina propria T(H)17 cell differentiation. Nature (2008) 455(7214):808–12. doi: 10.1038/nature07240 18716618

[18] Schenk U Frascoli M Proietti M Geffers R Traggiai E Buer J. ATP inhibits the generation and function of regulatory T cells through the activation of purinergic P2X receptors. Sci Signal (2011) 4(162):ra12. doi: 10.1126/scisignal.2001270 21364186

[19] Purvis HA Anderson AE Young DA Isaacs JD Hilkens CM. A negative feedback loop mediated by STAT3 limits human Th17 responses. J Immunol (2014) 193(3):1142–50. doi: 10.4049/jimmunol.1302467 24973454

[20] Li M Yang C Wang Y Song W Jia L Peng X. The expression of P2X7 receptor on Th1, Th17, and regulatory T cells in patients with systemic lupus erythematosus or rheumatoid arthritis and its correlations with active disease. J Immunol (2020) 205(7):1752–62. doi: 10.4049/jimmunol.2000222 32868411

[21] Labasi JM Petrushova N Donovan C McCurdy S Lira P Payette MM. Absence of the P2X7 receptor alters leukocyte function and attenuates an inflammatory response. J Immunol (2002) 168(12):6436–45. doi: 10.4049/jimmunol.168.12.6436 12055263

[22] Chen L Brosnan CF. Exacerbation of experimental autoimmune encephalomyelitis in *p2x7*R-/- mice: Evidence for loss of apoptotic activity in lymphocytes. J Immunol (2006) 176(5):3115–26. doi: 10.4049/jimmunol.176.5.3115 16493071

[23] Kurashima Y Amiya T Nochi T Fujisawa K Haraguchi T Iba H. Extracellular ATP mediates mast cell-dependent intestinal inflammation through P2X7 purinoceptors. Nat Commun (2012) 3:1034. doi: 10.1038/ncomms2023 22948816 PMC3658010

[24] Zhao J Wang H Dai C Zhang H Huang Y Wang S. *p2x7* blockade attenuates murine lupus nephritis by inhibiting activation of the NLRP3/ASC/caspase 1 pathway. Arthritis Rheumatol (2013) 65(12):3176–85. doi: 10.1002/art.38174 PMC4038356 24022661

[25] Faliti CE Gualtierotti R Rottoli E Gerosa M Perruzza L Romagnani A. *p2x7* receptor restrains pathogenic tfh cell generation in systemic lupus erythematosus. J Exp Med (2019) 216(2):317–36. doi: 10.1084/jem.20171976 PMC6363434 30655308

[26] Safya H Mellouk A Legrand J Le Gall SM Benbijja M Kanellopoulos-Langevin C. Variations in cellular responses of mouse T cells to adenosine-5’-Triphosphate stimulation do not depend on P2X7 receptor expression levels but on their activation and differentiation stage. Front Immunol (2018) 9:360. doi: 10.3389/fimmu.2018.00360 29535730 PMC5835135

[27] Mellouk A Bobé P. CD8(+), but not CD4(+) effector/memory T cells, express the CD44(high)CD45RB(high) phenotype with aging, which displays reduced expression levels of P2X7 receptor and ATP-induced cellular responses. FASEB J (2019) 33(3):3225–36. doi: 10.1096/fj.201800867R 30383448

[28] Le Gall SM Legrand J Benbijja M Safya H Benihoud K Kanellopoulos JM. Loss of P2X7 receptor plasma membrane expression and function in pathogenic B220+ double-negative T lymphocytes of autoimmune MRL/*lpr* mice. PloS One (2012) 7(12):e52161. doi: 10.1371/journal.pone.0052161 23284917 PMC3528777

[29] Bobé P Bonardelle D Benihoud K Opolon P Chelbi-Alix MK. Arsenic trioxide: A promising novel therapeutic agent for lymphoproliferative and autoimmune syndromes in MRL/*lpr* mice. Blood (2006) 108(13):3967–75. doi: 10.1182/blood-2006-04-020610 16926289

[30] Kinjyo I Gordon SM Intlekofer AM Dowdell K Mooney EC Caricchio R. Cutting edge: Lymphoproliferation caused by fas deficiency is dependent on the transcription factor eomesodermin. J Immunol (2010) 185(12):7151–5. doi: 10.4049/jimmunol.1003193 PMC2997140 21076068

[31] Qui HZ Hagymasi AT Bandyopadhyay S St Rose MC Ramanarasimhaiah R Menoret A. CD134 plus CD137 dual costimulation induces eomesodermin in CD4 T cells to program cytotoxic Th1 differentiation. J Immunol (2011) 187(7):3555–64. doi: 10.4049/jimmunol.1101244 PMC3178659 21880986

[32] Bride K Teachey D. Autoimmune lymphoproliferative syndrome: More than a FAScinating disease. F1000Res (2017) 6:1928. doi: 10.12688/f1000research.11545.1 29123652 PMC5668920

[33] Le Gallo M Poissonnier A Blanco P Legembre P. CD95/Fas, non-apoptotic signaling pathways, and kinases. Front Immunol (2017) 8:1216. doi: 10.3389/fimmu.2017.01216 29021794 PMC5623854

[34] Nolz JC Richer MJ. Control of memory CD8(+) T cell longevity and effector functions by IL-15. Mol Immunol (2020) 117:180–8. doi: 10.1016/j.molimm.2019.11.011 PMC6928418 31816491

[35] Zumwalt TJ Arnold M Goel A Boland CR. Active secretion of CXCL10 and CCL5 from colorectal cancer microenvironments associates with GranzymeB+ CD8+ T-cell infiltration. Oncotarget (2015) 6(5):2981–91. doi: 10.18632/oncotarget.3205 PMC4413778 25671296

[36] D’Asaro M Dieli F Caccamo N Musso M Porretto F Salerno A. Increase of CCR7- CD45RA+ CD8 T cells (T(EMRA)) in chronic graft-versus-host disease. Leukemia (2006) 20(3):545–7. doi: 10.1038/sj.leu.2404079 16408100

[37] Romero P Zippelius A Kurth I Pittet MJ Touvrey C Iancu EM. Four functionally distinct populations of human effector-memory CD8+ T lymphocytes. J Immunol (2007) 178(7):4112–9. doi: 10.4049/jimmunol.178.7.4112 17371966

[38] Jacob N Stohl W. Autoantibody-dependent and autoantibody-independent roles for b cells in systemic lupus erythematosus: past, present, and future. Autoimmunity (2010) 43(1):84–97. doi: 10.3109/08916930903374600 20014977 PMC2809122

[39] Teachey DT Seif AE Grupp SA. Advances in the management and understanding of autoimmune lymphoproliferative syndrome (ALPS). Br J Haematol (2010) 148(2):205–16. doi: 10.1111/j.1365-2141.2009.07991.x PMC2929682 19930184

[40] Tinhofer I Marschitz I Kos M Henn T Egle A Villunger A. Differential sensitivity of CD4+ and CD8+ T lymphocytes to the killing efficacy of fas (Apo-1/CD95) ligand+ tumor cells in b chronic lymphocytic leukemia. Blood (1998) 91(11):4273–81. doi: 10.1182/blood.V91.11.4273 9596676

[41] Peng SL Moslehi J Craft J. Roles of interferon-gamma and interleukin-4 in murine lupus. J Clin Invest. (1997) 99(8):1936–46. doi: 10.1172/JCI119361 PMC508018 9109438

[42] Tang W Wang H Tian R Saret S Cheon H Claudio E. Bcl-3 inhibits lupus-like phenotypes in BL6/*lpr* mice. Eur J Immunol (2020) 51(1):197–205. doi: 10.1002/eji.202048584 32652549

[43] Low JT Hughes P Lin A Siebenlist U Jain R Yaprianto K. Impact of loss of NF-kappaB1, NF-kappaB2 or c-REL on SLE-like autoimmune disease and lymphadenopathy in fas(*lpr*/*lpr*) mutant mice. Immunol Cell Biol (2016) 94(1):66–78. doi: 10.1038/icb.2015.66 26084385

[44] Boggio E Clemente N Mondino A Cappellano G Orilieri E Gigliotti CL. IL-17 protects T cells from apoptosis and contributes to development of ALPS-like phenotypes. Blood (2014) 123(8):1178–86. doi: 10.1182/blood-2013-07-518167 24363402

[45] Kyttaris VC Zhang Z Kuchroo VK Oukka M Tsokos GC. Cutting edge: IL-23 receptor deficiency prevents the development of lupus nephritis in C57BL/6-*lpr*/*lpr* mice. J Immunol (2010) 184(9):4605–9. doi: 10.4049/jimmunol.0903595 PMC2926666 20308633

[46] Zhang Z Kyttaris VC Tsokos GC. The role of IL-23/IL-17 axis in lupus nephritis. J Immunol (2009) 183(5):3160–9. doi: 10.4049/jimmunol.0900385 PMC2766304 19657089

[47] Waldmann TA Miljkovic MD Conlon KC. Interleukin-15 (dys)regulation of lymphoid homeostasis: Implications for therapy of autoimmunity and cancer. J Exp Med (2020) 217(1):e20191062. doi: 10.1084/jem.20191062 31821442 PMC7037239

[48] Inoue S Unsinger J Davis CG Muenzer JT Ferguson TA Chang K. IL-15 prevents apoptosis, reverses innate and adaptive immune dysfunction, and improves survival in sepsis. J Immunol (2010) 184(3):1401–9. doi: 10.4049/jimmunol.0902307 PMC2937828 20026737

[49] Goel RR Wang X O’Neil LJ Nakabo S Hasneen K Gupta S. Interferon lambda promotes immune dysregulation and tissue inflammation in TLR7-induced lupus. Proc Natl Acad Sci U S A. (2020) 117(10):5409–19. doi: 10.1073/pnas.1916897117 PMC7071891 32094169

[50] Sanjabi S Oh SA Li MO. Regulation of the immune response by TGF-β: From conception to autoimmunity and infection. Cold Spring Harb Perspect Biol (2017) 9(6). doi: 10.1101/cshperspect.a022236 PMC5453394 28108486

[51] Veldhoen M. Interleukin 17 is a chief orchestrator of immunity. Nat Immunol (2017) 18(6):612–21. doi: 10.1038/ni.3742 28518156

[52] Okazaki H Hirata D Kamimura T Sato H Iwamoto M Yoshio T. Effects of FTY720 in MRL-*lpr*/*lpr* mice: Therapeutic potential in systemic lupus erythematosus. J Rheumatol (2002) 29(4):707–16. 11950011

[53] Lai ZW Kelly R Winans T Marchena I Shadakshari A Yu J. Sirolimus in patients with clinically active systemic lupus erythematosus resistant to, or intolerant of, conventional medications: A single-arm, open-label, phase 1/2 trial. Lancet (2018) 391(10126):1186–96. doi: 10.1016/S0140-6736(18)30485-9 PMC5891154 29551338

[54] Volkl S Rensing-Ehl A Allgauer A Schreiner E Lorenz MR Rohr J. Hyperactive mTOR pathway promotes lymphoproliferation and abnormal differentiation in autoimmune lymphoproliferative syndrome. Blood (2016) 128(2):227–38. doi: 10.1182/blood-2015-11-685024 27099149

[55] Mao Y van Hoef V Zhang X Wennerberg E Lorent J Witt K. IL-15 activates mTOR and primes stress-activated gene expression leading to prolonged antitumor capacity of NK cells. Blood (2016) 128(11):1475–89. doi: 10.1182/blood-2016-02-698027 PMC5025899 27465917

[56] Gabriel SS Tsui C Chisanga D Weber F Llano-Leon M Gubser PM. Transforming growth factor-beta-regulated mTOR activity preserves cellular metabolism to maintain long-term T cell responses in chronic infection. Immunity (2021) 54(8):1698–1714 e5. doi: 10.1016/j.immuni.2021.06.007 34233154

[57] Bian S Sun X Bai A Zhang C Li L Enjyoji K. *p2x7* integrates PI3K/AKT and AMPK-PRAS40-mTOR signaling pathways to mediate tumor cell death. PloS One (2013) 8(4):e60184. doi: 10.1371/journal.pone.0060184 23565201 PMC3615040

