# Supplementary material for: P2X7 purinergic receptor plays a critical role in maintaining T-cell homeostasis and preventing lupus pathogenesis
Source: Front Immunol. 2022 Sep 29;13:957008. doi: 10.3389/fimmu.2022.957008 (PMC9556828; doi:10.3389/fimmu.2022.957008)
Supplement: Supplementary file 1 [file DataSheet_1.docx]

Supplementary Material

# Supplementary Figures and Tables

## Supplementary Figures


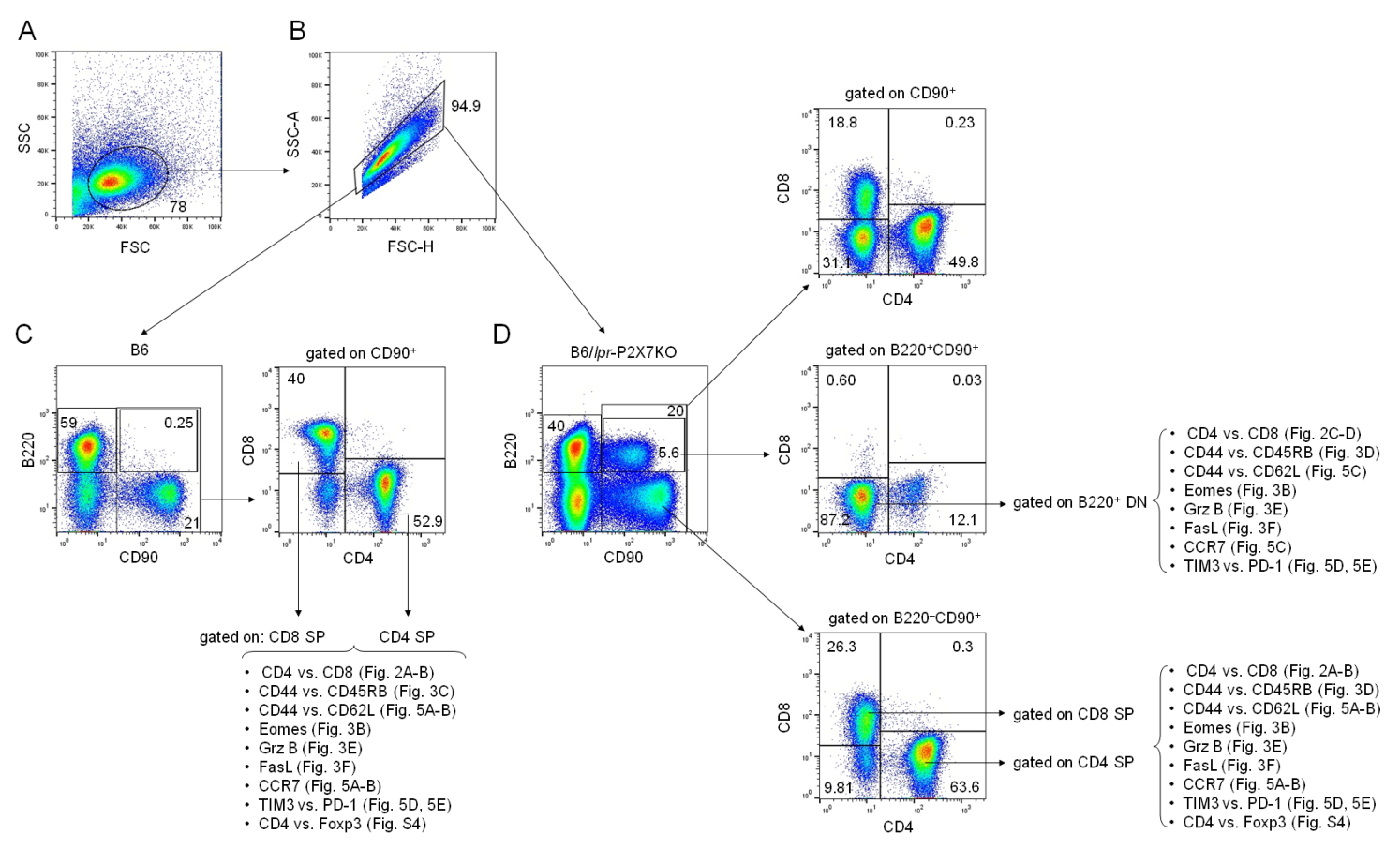


**Figure S1.** **Representative dot plot showing sequential gating for identifying the different CD4 or CD8 single-positive T cells and B220^+^ CD4^–^CD8^–^ double-negative T-cell subsets**. Spleen cells from B6 and B6/*lpr*-P2X7KO mice were stained with either fluorescent mAbs against phenotypic markers CD90, B220, CD4, and CD8 or isotype controls and analyzed by flow cytometry. FSC vs. SSC dot plots were used to select single, viable cells and exclude debris, dead cells and doublets. **CD90 vs. B220 dot plot** was used to **select B220^–^CD90^+^ and B220^+^CD90^+^ T cells as well as B220^+^CD90^–^B cells in the FSC^hi^SSC^lo^-gated cell population. The B220^–^CD90^+^ and B220^+^CD90^+^ T cells** were then further gated by the expression of CD4 **and CD8**. At least 20 000 events were analyzed from each sample. Subsequent analyses on the relative expression of CD45RB, CD44, CD62L, CCR7, Eomes, Granzyme (Grz) B, membrane Fas ligand (FasL), TIM3 and PD-1 in **B220^–^CD90^+^ (gated on CD4 SP or CD8 SP) and B220^+^ CD4^–^CD8^–^ CD90^+^ (gated on B220^+^ DN) T cells are displayed in Figures mentioned in parentheses.**


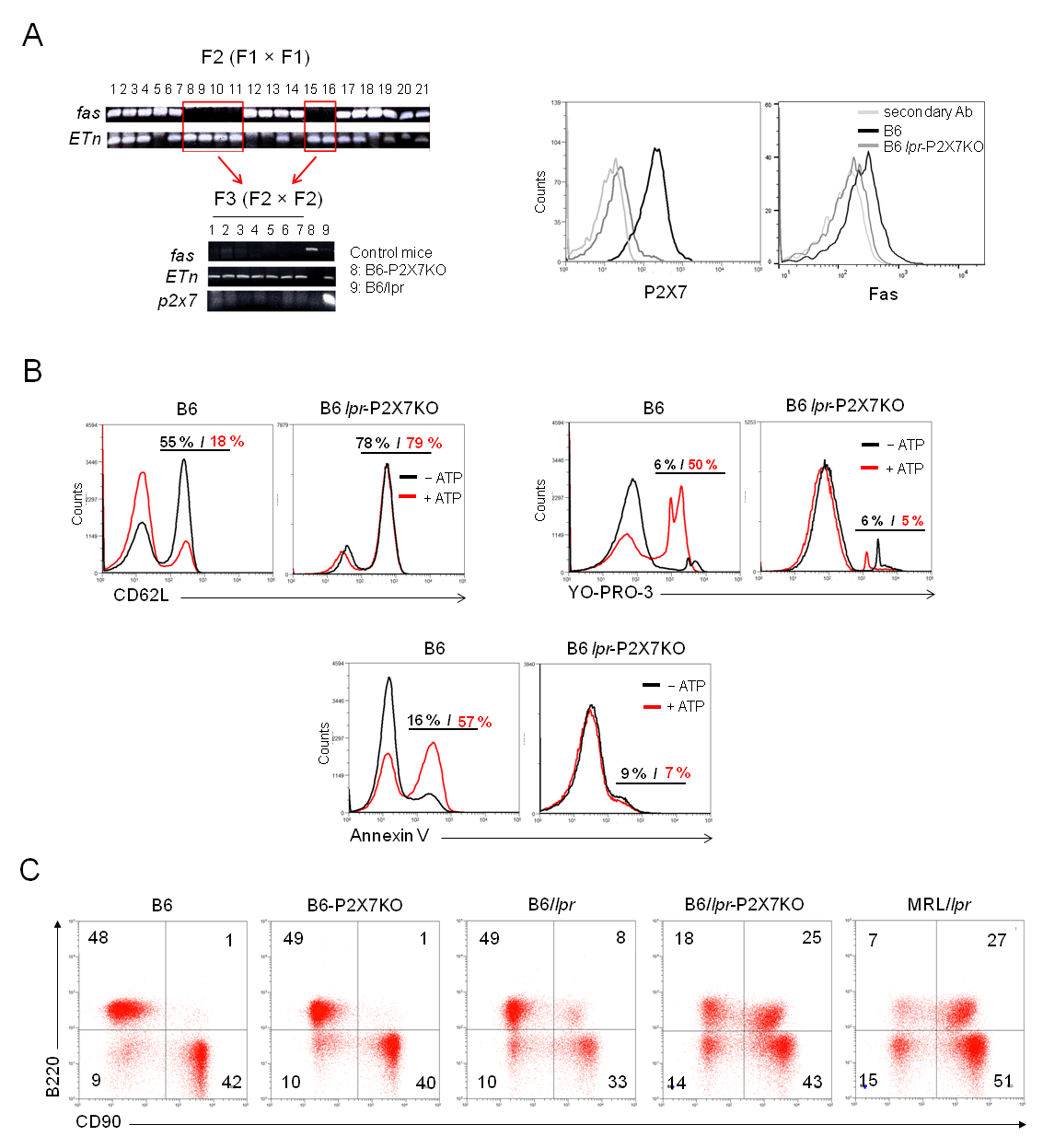


**Figure S2. (A) Generation of Fas and P2X7 deficient B6/*lpr-*P2X7KO mice by conventional crosses between B6/*lpr* and B6-P2X7KO mice, and flow cytometry analysis of the membrane protein expression levels.** Fas-deficient **(**B6/*lpr*) and P2X7-deficient (B6-P2X7KO) B6 mice were mated to produce F1 mice that are uniformly heterozygous for the *fas* and *p2x7* mutations. F1 mice were subsequently mated to produce F2 mice. Screening of mice status for both Fas and P2X7 expression was assessed at DNA and protein levels by PCR and flow cytometry, respectively. (**A, left panels**) Wild-type and mutant alleles of the *p2x7* and *fas* genes were screened by PCR using genomic DNA extracted from tails of F2 and F3 generation mice as well as forward and reverse primers for the *p2x7* gene (246 pb amplicon), the *fas* gene (179 pb amplicon) and for *ETn* insertion (*lpr* mutation) into the *fas* gene (217 pb amplicon). (**A, right panels**) Levels of Fas and P2X7 membrane expression on CD90^+^ T cells from B6 and B6/*lpr*-P2X7KO mice were measured using fluorescent anti-Fas mAb as well as rabbit polyclonal anti-P2X7 antiserum (1:100) and fluorescent-conjugated goat anti-rabbit IgG F(ab)’_2_ secondary Ab. **(B) ATP-mediated cellular responses in CD90^+^ T cells.** Spleen cells from B6 and B6/*lpr*-P2X7KO mice were either left unstimulated or stimulated with 500 µM ATP for 30 min in the presence or absence of YO-PRO-3 fluorescent probe. Cells were subsequently stained with fluorescent antibodies against phenotypic markers CD90, B220 and CD62L as well as Annexin V fluorescent probe. CD62L shedding, pore formation, or PS exposure were assessed by flow cytometry on gated CD90^+^ T cells. Results on CD62L shedding, pore formation, or PS exposure are expressed as the mean percentage of CD62L^+^, YO-PRO-3^+^ or Annexin V^+^ cells with (red lines) or without (black lines) ATP treatment. Data are representative of at least six independent experiments with 7 mice per group per experiment. **(C)** **Flow cytometric quantification of splenic T and B cell populations.** Spleen cells from 7- to 8-month-old B6, B6-P2X7KO, B6*/lpr* and B6*/lpr-P2X7KO* and 4-month-old MRL*/lpr* mice were stained with anti-CD90 and anti-B220 mAb to assess the percentages of CD90^+^ T cells (either B220^‒^ or B220^+^) and B220^+^CD90^‒^ B cells. Dot plots are representative of at least six independent mice per strain.


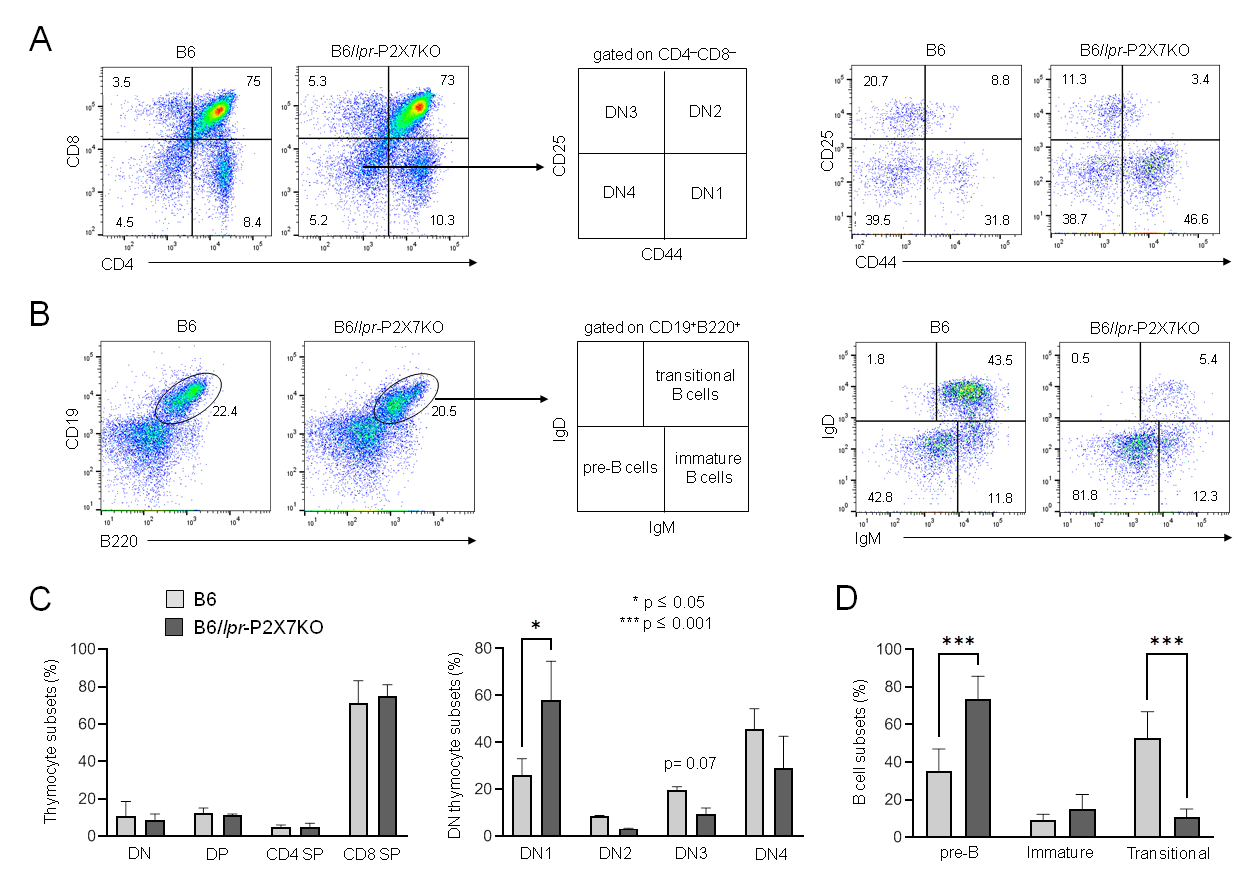


**Figure S3. Phenotype of thymocytes and bone marrow B cells in B6/*lpr*-P2X7KO mice.** **(A)** Thymocytes from 6- to 7-month-old wild-type B6 and B6/*lpr*-P2X7KO mice were stained with either fluorescent mAbs against phenotypic markers CD4, CD8, CD25 and CD44 or isotype controls. CD44 versus CD25 expression was assessed within the thymic CD4^‒^CD8^‒^ cells by flow cytometry. **(B)** Bone marrow cells from 6- to 7-month-old wild-type B6 and B6/*lpr*-P2X7KO mice were stained with either fluorescent mAbs against phenotypic markers IgM, IgD, CD19 and B220 or isotype controls. IgM versus IgD expression was assessed within the CD19^+^B220^+^ cells by flow cytometry. **(C–D)** Bar graphs show the mean percentage (SD, n = 3 mice per strain) of bone marrow B cell and thymocyte subsets from wild-type B6 and B6/*lpr*-P2X7KO mice. Asterisks denote statistically significant differences between the indicated groups (* p ≤ 0.05; *** p ≤ 0.001).


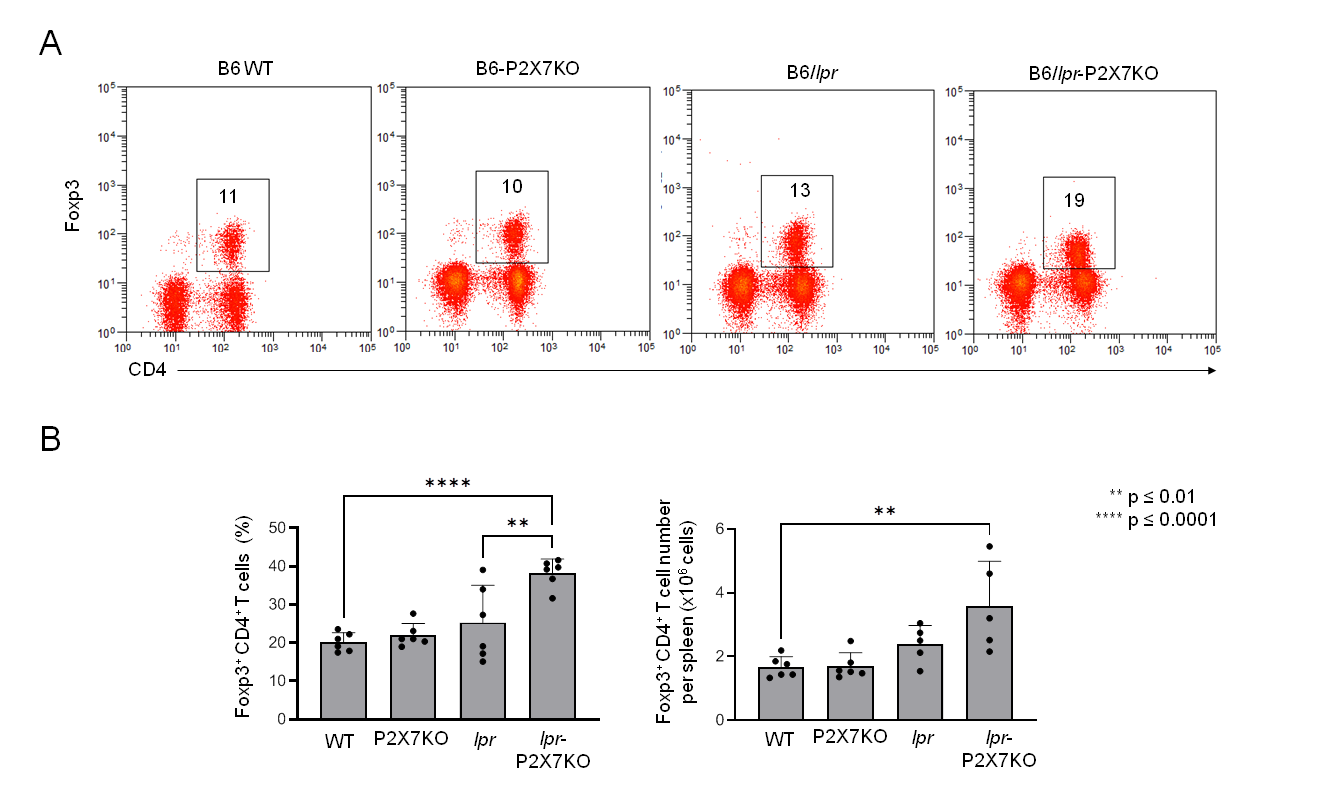


**Figure S4. Accumulation of Foxp3^+^ Tregs in the spleen of B6*/lpr-*P2X7KO mice.** Spleen cells from 7- to 8-month-old female B6, B6-P2X7KO, B6/*lpr* and B6/*lpr*-P2X7KO mice were stained with either fluorescent mAbs against phenotypic markers CD90, B220, CD4, CD8, and Foxp3 or isotype controls. (**A**) CD4 versus Foxp3 expression was assessed by flow cytometry to quantify percentages of Foxp3^+^CD4^+^ regulatory T cells. Dot plots are representative of at least six independent mice per group. (**B**) Bar graphs show the percentage and absolute number of Foxp3^+^CD4^+^ regulatory T cells per spleen. Data are expressed as mean (SD) of 5-6 mice per strain, and each dot represents an individual mouse. Error bars in graphs show SD. Horizontal lines with asterisks indicate statistically significant differences assessed with unpaired two-tailed *t*-test. Asterisks denote statistically significant differences between the indicated groups (** p ≤ 0.01, **** p ≤ 0.0001).

## Supplementary Tables

Table S1. Cytokine levels (pg/ml) in sera from B6/*lpr*-P2X7KO mice, parental strains and MRL/*lpr* mice

|  | **B6** | **B6-P2X7KO** | **B6/*lpr*** | **B6/*lp*r-P2X7KO** | **MRL/*lpr*** |
| --- | --- | --- | --- | --- | --- |
| **IL-1α** | 6.45 (4.2)* | 10.38 (5.4)* | 15.64 (6.5)* | 6.83 (5.1)* | 19.34 (41.6)* |
| **IL-2** | 0 | 0.32 (0.83) | 17.5 (23.8) | 34.97 (60.9) | 5.83 (13.4) |
| **IL-3** | 0.23 (0.22) | 0.22 (0.15) | 1.27 (1.05) | 4.55 (6.4) | 0.32 (0.3) |
| **IL-4** | 2.27 (1.7) | 7.84 (6.7) | 2.56 (1.6) | 10.75 (14.6) | 5.92 (4.3) |
| **IL-5** | 45.5 (25.2) | 64.25 (49.4) | 51.8 (33.7) | 61.58 (52.3) | 29.99 (18.2) |
| **IL-9** | 30.45 (52) | 17.89 (22.2) | 53.08 (63) | 10.89 (10.4) | 7.76 (8.5) |
| **IL-12p70** | 7.92 (9.6) | 4.8 (1.9) | 34.4 (34.2) | 170.8 (293) | 14.17 (22.3) |
| **IL-13** | 5.97 (7.4) | 9.95 (8.3) | 8.61 (10.2) | 12.84 (8.8) | 7.7 (5.8) |
| **IL-18** | 569 (386) | 1738 (1338) | 583 (601) | 2165 (3396) | 880 (778) |
| **IL-22** | 65 (29.7) | 375.3 (435) | 82.39 (59) | 360 (368) | 48.5 (52.7) |
| **IL-27** | 50 (47) | 70.44 (94) | 70.05 (53) | 28.7 (18.5) | 47.26 (23.5) |
| **IL-31** | 0 | 0 | 22.2 (27.2) | 37.45 (61) | 16.46 (54.6) |
| **IL-33** | 0 | 0 | 90 (155) | 122 (154) | 348.25(743) |
| **IFN-α** | 0 | 0 | 6.43 (15.5) | 4.9 (14.7) | 0 (0) |
| **GM-CSF** | 2.8 (3.5) | 28.99 (34) | 3.71 (3.7) | 31.32 (38.5) | 13.96 (31.6) |
| **G-CSF** | 10.08 (5.9) | 13.36 (11.8) | 61.1 (54.5) | 63.7 (96) | 15.3 (22.4) |
| **M-CSF** | 0.03 (0.05) | 0.06 (0.13) | 0.48 (0.68) | 1.4 (3.4) | 0.49 (0.75) |
| **LIF** | 3.88 (3.87) | 5.03 (2.99) | 14.5 (16.4) | 6.57 (5.02) | 8.25 (11.5) |
| **CCL2/MCP-1** | 62.84 (61) | 8.76 (19.5) | 57 (56.6) | 29.6 (47.5) | 89.13 (80.7) |
| **CCL3/MIP-1α** | 18.79 (7.4) | 21.95 (3.01) | 23 (10.8) | 22.05 (2.9) | 28.34 (7.8) |
| **CCL4/MIP-1** | 10.65 (3.6) | 10.95 (1.4) | 28.3 (20.2) | 48.75 (49.9) | 26.46 (11.7) |
| **CCL5/RANTES** | 74.96 (18) | 99.61 (43.4) | 78.8 (28.7) | 94.76 (30.6) | 166.94 (88.8) |
| **CCL7/MCP-3** | 190.5 (36) | 421.52 (100) | 231.7 (89) | 319 (143) | 444.84 (193) |
| **CCL11/Eotaxin** | 1136 (387) | 1256 (239) | 1214 (281) | 850 (354) | 979 (174) |
| **CXCL1/GRO-α** | 86.5 (44.4) | 173.98 (118) | 74.37 (32) | 247 (321) | 169.77 (140) |
| **CXCL2/MIP-2** | 22.75 (4.5) | 23.33 (3.6) | 23.99 (2.7) | 24.97 (9.8) | 27.06 (15.7) |
| **CXCL5/ENA-78** | 3039 (725) | 3264 (853) | 2599 (917) | 2040 (649) | 1252 (344) |

* SDs are given in parentheses, n = 5-11 mice per strain.
